# MSC Membrane‐Coated circPROSC‐siRNA Nanoparticles for Ameliorating Craniosynostosis by Inhibiting Premature Suture Ossification

**DOI:** 10.1002/advs.202510454

**Published:** 2025-11-03

**Authors:** Zhenkun Weng, Xiu Chen, Jin Xu, Qing Yan, Jian Jiao, Qian Liu, Aihua Gu

**Affiliations:** ^1^ State Key Laboratory of Reproductive Medicine and Offspring Health School of Public Health Nanjing Medical University Nanjing 211166 P. R. China; ^2^ Jiangsu Environmental Health Risk Assessment Engineering Research Center Key Laboratory of Modern Toxicology of Ministry of Education Center for Global Health Nanjing Medical University Nanjing 211166 P. R. China; ^3^ Department of Neurosurgery Children's Hospital of Nanjing Medical University Nanjing 211166 P. R. China

**Keywords:** craniosynostosis, membrane‐coated nanoparticles, osteogenic differentiation, siRNA delivery

## Abstract

Craniosynostosis is a congenital craniofacial disorder caused by excessive osteogenic differentiation of mesenchymal stem cells (MSCs) within cranial sutures. Due to incompletely understood regulatory mechanisms, effective solutions for early diagnosis and minimally invasive therapy remain lacking. In this study, plsama circPROSC is first identified as an independent risk factor for craniosynostosis. Through MSCs osteogenic differentiation and nude mouse ectopic bone formation assays, circPROSC promotes osteogenesis via miR‐6815‐5p‐mediated modulation of the Wnt signaling pathway. Furthermore, MSC membrane‐coated siRNA nanoparticles (MM@Lipo/siRNA) targeting circPROSC (si‐circPROSC) have been developed to inhibit its expression in the coronal suture by postnatal cranial microinjection, which attenuated premature coronal suture closure in a craniosynostosis mouse model. Collectively, this study provides the first evidence that circPROSC is a promising diagnostic marker and a potential therapeutic target for craniosynostosis.

## Graphical abstract

CircPROSC promotes craniosynostosis by sponging miR‐6815‐5p, thereby activating WNT3A/β‐catenin signaling and enhancing RUNX2‐mediated osteogenesis. Targeted silencing of circPROSC with MSC membrane–coated siRNA nanoparticles (MM@Lipo/si‐circPROSC) delivered postnatally into cranial sutures attenuates premature suture closure in Twist1^+/–^ mice, highlighting circPROSC as a novel diagnostic biomarker and therapeutic target.

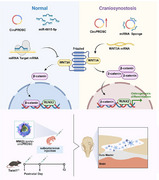

## 1 Introduction

Craniosynostosis, a significant congenital craniofacial disorder, results from the premature fusion of cranial sutures in infants due to abnormal intramembranous ossification.^[^ ^1^, ^2^ ^]^ This condition leads to distinct head shape abnormalities and severe symptoms, such as elevated intracranial pressure, restricted brain growth, and neurological deficits.^[^ ^3^ ^]^ The prevalence of craniosynostosis is ≈7 in 10 000 live births, with reports suggesting an increasing incidence.^[^ ^4^ ^]^ Several genes, including TWIST1/2, FGFR1/2/3, and MSX2, have been confirmed to play a role in the pathogenesis of craniosynostosis.^[^ ^5^, ^6^ ^]^ However, epidemiological studies have shown that ≈70% of patients lack identifiable pathogenic mutations.^[^ ^7^ ^]^ In addition, monozygotic twin studies have demonstrated phenotypic variability and discordance in craniosynostosis, even among individuals with identical genotypes.^[^ ^8^, ^9^ ^]^ These findings further suggest that genetic factors alone do not fully explain the disease and highlight the significance of epigenetic regulation in its pathogenesis.

Emerging evidence highlights the epigenetic factors that influence the pathogenesis of craniosynostosis,^[^ ^10^, ^11^ ^]^ although the specific regulatory mechanisms remain largely unclear. The upregulation of H3K27me3 demethylases has been linked to the development of the craniosynostosis phenotype, and the long non‐coding RNA (lncRNA) HOTAIR may contribute to osteoclast formation and the onset of craniosynostosis.^[^ ^12^, ^13^ ^]^ Circular RNAs (circRNAs) have emerged as promising biomarkers for disease diagnosis and progression due to their high stability and tissue specificity.^[^ ^14^, ^15^ ^]^ In fact, circRNAs have been implicated in regulating various bone diseases, such as osteoporosis, osteoarthritis and intervertebral disc degeneration.^[^ ^16^, ^17^, ^18^ ^]^ For instance, circSTX12 contributes to osteoporosis by inhibiting osteogenesis through competitively binding to the E3 ubiquitin ligase CBL.^[^ ^19^ ^]^ CircRNA.33186 is involved in the pathogenesis of osteoarthritis by directly binding to miR‐127‐5p and inhibiting its activity, thereby increasing the expression of MMP‐13.^[^ ^20^ ^]^ Furthermore, the downregulation of circSMAD4 promotes cartilage endplate ossification by causing Yap1 mRNA degradation, which leads to intervertebral disc degeneration.^[^ ^21^ ^]^ Given that research on the regulatory role of circRNAs in craniosynostosis remains limited, investigating this role becomes not only crucial for understanding the epigenetic basis of this disorder but also holds the key to developing innovative diagnostic and treatment strategies.

Some key challenges to the study of craniosynostosis include its low incidence, which results in a lack of large‐scale population data, and the early age of onset, which makes it difficult to identify early pathogenic targets and increases the likelihood of missing the optimal time for intervention. To date, surgical correction remains the primary treatment for craniosynostosis, and minimally invasive therapies are still underdeveloped.^[^ ^22^, ^23^ ^]^ It has been reported that combining the implantation of MSCs with biodegradable materials supports cranial suture regeneration, and pharmacological interventions have also shown improved treatment outcomes.^[^ ^24^, ^25^, ^26^ ^]^ Despite these advances, current therapeutic progress remains unsatisfactory due to the lack of effective targets that can both provide early warning and enable early intervention to prevent disease progression. Therefore, there is an urgent need to identify novel targets that can improve the treatment of craniosynostosis.

RNA interference (RNAi) holds great promise for various therapeutic strategies due to its ability to specifically silence genes.^[^ ^27^ ^]^ However, the clinical application of RNAi is hindered by several challenges, including the limited bioavailability of siRNA, its susceptibility to enzymatic degradation, short half‐life, off‐target effects, poor cellular uptake, and potential immunogenicity.^[^ ^28^ ^]^ Nanoparticles (NPs) demonstrate high biocompatibility and are effective in diagnostics, bioimaging, and precision therapy, making the integration of NPs with siRNA a promising approach.^[^ ^29^ ^]^ Surface characterization of NPs is essential, as those with unsuitable surface properties are recognized and cleared by the immune system after injection.^[^ ^30^ ^]^ To address this limitation, a biomimetic cell membrane camouflaging strategy has been incorporated into nano‐drug delivery systems, endowing the nanosystem with novel properties, such as immune evasion and targeted disease treatment.

In this study, we identified a specific molecular biomarker for craniosynostosis, circPROSC, which is significantly elevated in patients with craniosynostosis and may serve as a promising diagnostic marker. CircPROSC promotes osteogenic differentiation, which leads to craniosynostosis, through miR‐6815‐5p‐mediated modulation of the Wnt signaling pathway. We developed a novel nanoparticle system composed of MSC membranes and cationic liposomes for the targeted delivery of circPROSC siRNA into cranial sutures, which effectively alleviated premature coronal suture closure in a craniosynostosis mouse model.

## 2 Results

### 2.1 CircPROSC Expression is Upregulated in Craniosynostosis and Associated with hMSCs Osteogenesis

**Figure 1:**
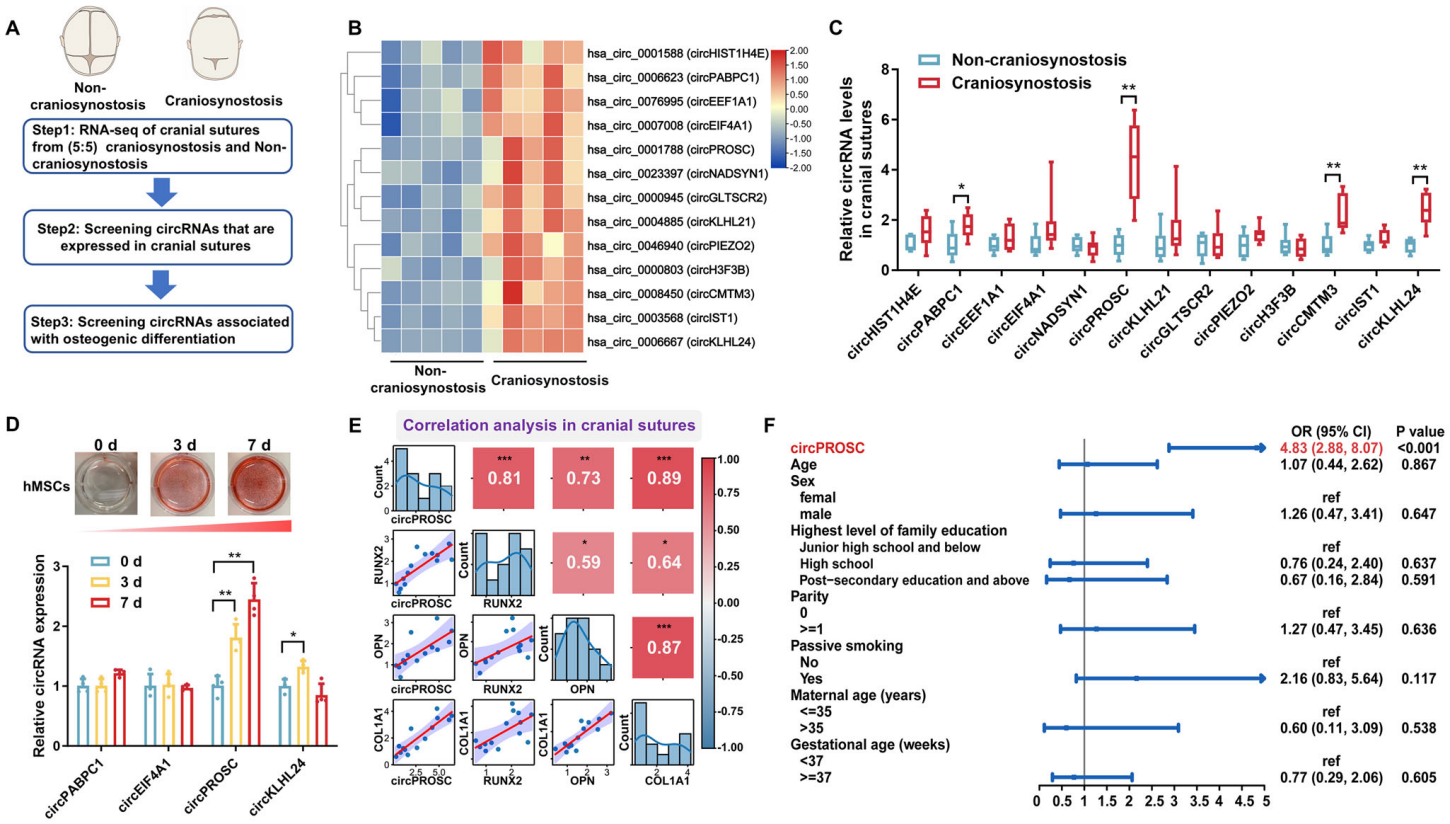
CircPROSC expression is upregulated in craniosynostosis and associated with hMSCs osteogenesis. A) Flowchart of the steps for screening circRNAs. B) Cluster heatmap of upregulated differentially expressed circRNAs (*n*  =  5). C) Levels of circRNAs in the cranial sutures of non‐craniosynostosis (*n*  =  6) and craniosynostosis patients (*n*  =  8). D) Dynamic expression of circRNAs during hMSCs osteogenesis at days 0, 3, and 7 (*n* = 4). E) Correlation analysis between circPROSC and osteogenic marker genes in cranial sutures. F) Odds ratios and 95% confidence intervals for associations between circPROSC expression and craniosynostosis risk. The results were adjusted for age, sex, family education level, parity, passive smoking, maternal age, and gestational age. Data are represented as the means±SD. Statistical significance was determined by a two‐tailed unpaired t‐test (C) and by one‐way ANOVA (D). *p < 0.05 and **p < 0.01.

The flowchart of the steps for identifying functional circRNAs involved in craniosynostosis is presented in **Figure** **1**A. The circRNA expression profiles in the cranial suture tissues of craniosynostosis patients (*n* = 5) and matched non‐craniosynostosis individuals (*n* = 5) were determined using a circRNA microarray. A total of 13 upregulated circRNAs were identified in cranial suture tissues from craniosynostosis patients (fold change, FC ≥ 2; FDR < 0.05) (Figure 1B; Table S1, Supporting Information). To validate the expression of selected circRNAs, we analyzed an independent set of cranial suture tissue samples from 8 craniosynostosis patients and 6 control subjects using RT‐qPCR. This analysis confirmed the upregulation of circPABPC1 (hsa_circ_0006623), circPROSC (hsa_circ_0001788), circCMTM3 (hsa_circ_0008450), and circKLHL24 (hsa_circ_0006667) (Figure 1C). Notably, circPROSC was the only circRNA that gradually increased during hMSCs osteogenic differentiation (Figure 1D). Further correlation analysis revealed that circPROSC expression was positively correlated with the expression of osteogenic markers (RUNX2, OPN, and COL1A1), which were significantly elevated in fused cranial suture tissues from craniosynostosis patients (Figure 1E; Figure S2, Supporting Information). Additionally, plasma circPROSC expression was associated with an increased risk of craniosynostosis (OR = 4.83; 95% CI: 2.88, 8.07) (Figure 1F). Furthermore, plasma circPROSC levels remained stable after repeated freeze–thaw cycles and under various durations of room‐temperature storage (Figure S3A,B, Supporting Information). CircPROSC exhibited good diagnostic accuracy for craniosynostosis, with an area under the curve (AUC) of 0.885 (Figure S3C, Supporting Information). These results indicate that circPROSC is positively correlated with osteogenesis and can serve as a potential biomarker for craniosynostosis.

### 2.2 CircPROSC Enhances the Osteogenic Capability of hMSCs

**Figure 2:**
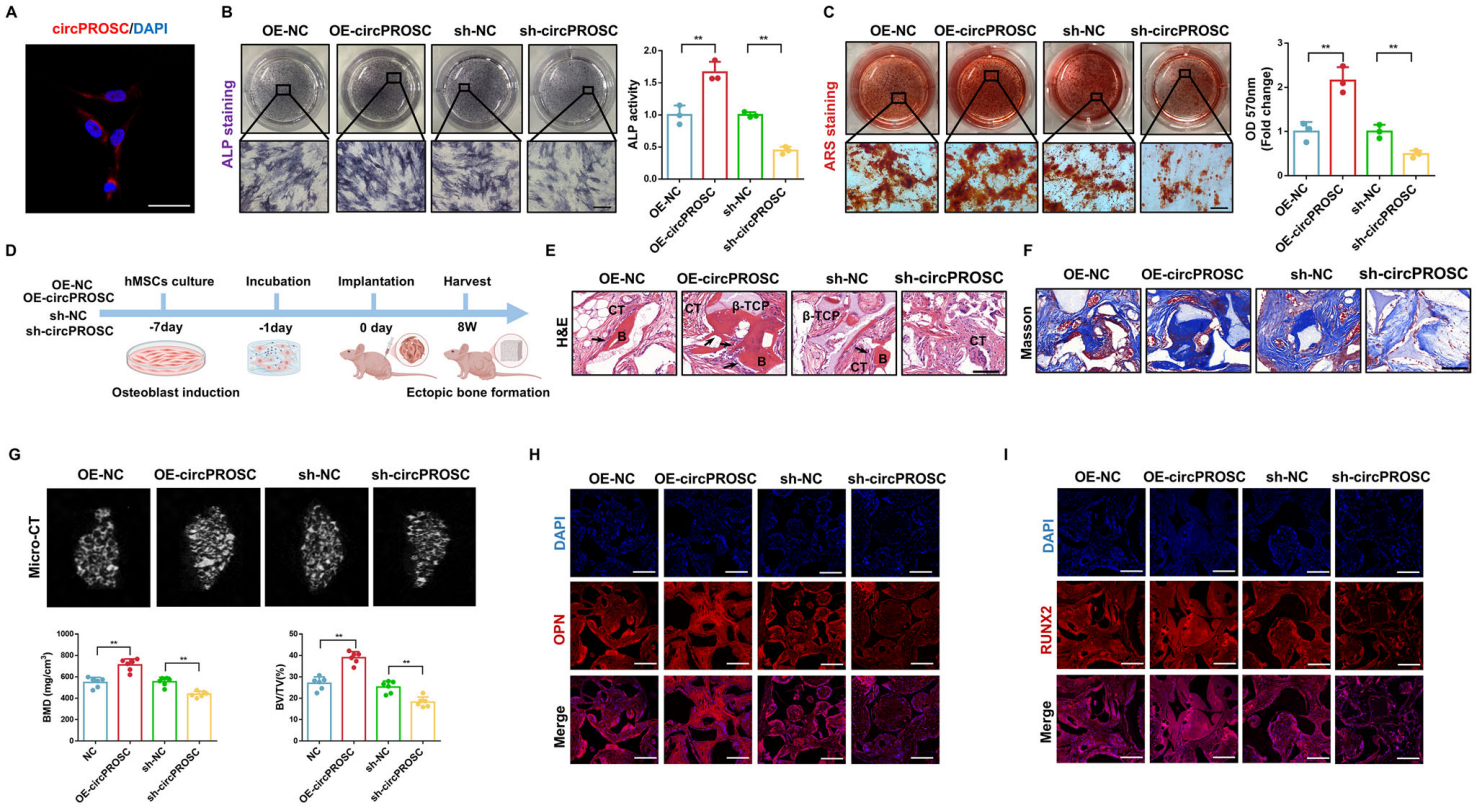
CircPROSC enhances the osteogenic capability of hMSCs. A) RNA fluorescence in situ hybridization of circPROSC. Nuclei were stained with DAPI; Scale bar = 50 µm. B) ALP staining and ALP activity of hMSCs were assessed on day 7 after osteoblast differentiation. Scale bar = 200 µm. C) Alizarin Red staining of hMSCs was performed on day 14 after osteoblast differentiation and semiquantitative analysis of ARS staining was conducted. Scale bar = 200 µm, D) Schematic representation of the heterotopic bone formation procedure. E) H&E staining and Masson's trichrome staining F) of the OE‐NC, OE‐circPROSC, sh‐NC, and sh‐circPROSC groups (B, bone; CT, connective tissue). The black arrows indicate the bone‐like structures. Scale bar = 100 µm. G) Micro‐CT 3D imaging of the tissue‐engineered bone constructs and quantitative analysis of bone mineral density (BMD) and bone volume fraction (BV/TV) of the newly formed bone. H) Immunofluorescence staining of OPN and I) RUNX2 in bone tissue samples. Scale bar = 50 µm. Data are represented as the means±SD, *n*  =  6 biologically independent mice. Statistical significance was determined by a two‐tailed unpaired t‐test. *p < 0.05 and **p < 0.01.

CircPROSC is derived from exon 2–4 circularization of the PROSC gene located on chr8 (37623043‐37623873) with a “head‐to‐tail” back‐spliced junction site, as verified by Sanger sequencing (Figure S4A, Supporting Information). Using cDNA and gDNA from hMSCs as templates, gel electrophoresis revealed that circPROSC was amplified with divergent primers only in cDNA, with no amplification observed in gDNA (Figure S4B, Supporting Information). In line with fluorescence in situ hybridization (FISH) (**Figure** **2**A), nuclear and cytoplasmic separation tests further validated that circPROSC was predominantly located in the cytoplasm (Figure S4C, Supporting Information). Moreover, circPROSC was resistant to RNase R digestion (which degrades linear RNA), whereas linear PROSC mRNA was significantly reduced after RNase R treatment (Figure S4D, Supporting Information). To assess the biological functions of circPROSC in hMSCs, we overexpressed and knocked down circPROSC and examined the osteogenic potential of hMSCs (Figure S5A, Supporting Information). ALP staining, ALP activity assay, and ARS staining showed that circPROSC overexpression significantly enhanced osteogenesis in hMSCs, whereas circPROSC knockdown had the opposite effect (Figure 2B,C). The expression of osteogenesis‐related proteins, including RUNX2, OPN and COL1A1, was distinctly upregulated by circPROSC overexpression and decreased by circPROSC knockdown (Figure S5B–D, Supporting Information).

To investigate the impact of circPROSC on hMSCs osteogenesis in vivo, hMSCs overexpressing circPROSC and loaded with β‐TCP composites were subcutaneously transplanted into BALB/c nude mice (Figure 2D). H&E and Masson's trichrome staining showed that circPROSC overexpression resulted increased bone‐like structures and collagen deposition, whereas circPROSC knockdown had the opposite effect (Figure 2E,F). Micro‐CT analysis showed that the bone mineral density (BMD) and bone volume fraction (BV/TV) were significantly higher in the circPROSC overexpression group, and lower in the circPROSC‐knockdown group (Figure 2G). Immunofluorescence analysis revealed that bone tissue with circPROSC overexpression exhibited increased levels of RUNX2 and OPN compared to the control group, while circPROSC knockdown showed the opposite trend (Figure 2H,I; Figure S6, Supporting Information). These results demonstrate that circPROSC promotes the osteogenic differentiation of hMSCs both in vitro and in vivo.

### 2.3 CircPROSC Facilitates the Osteogenic Differentiation of hMSCs by Regulating WNT3A

**Figure 3:**
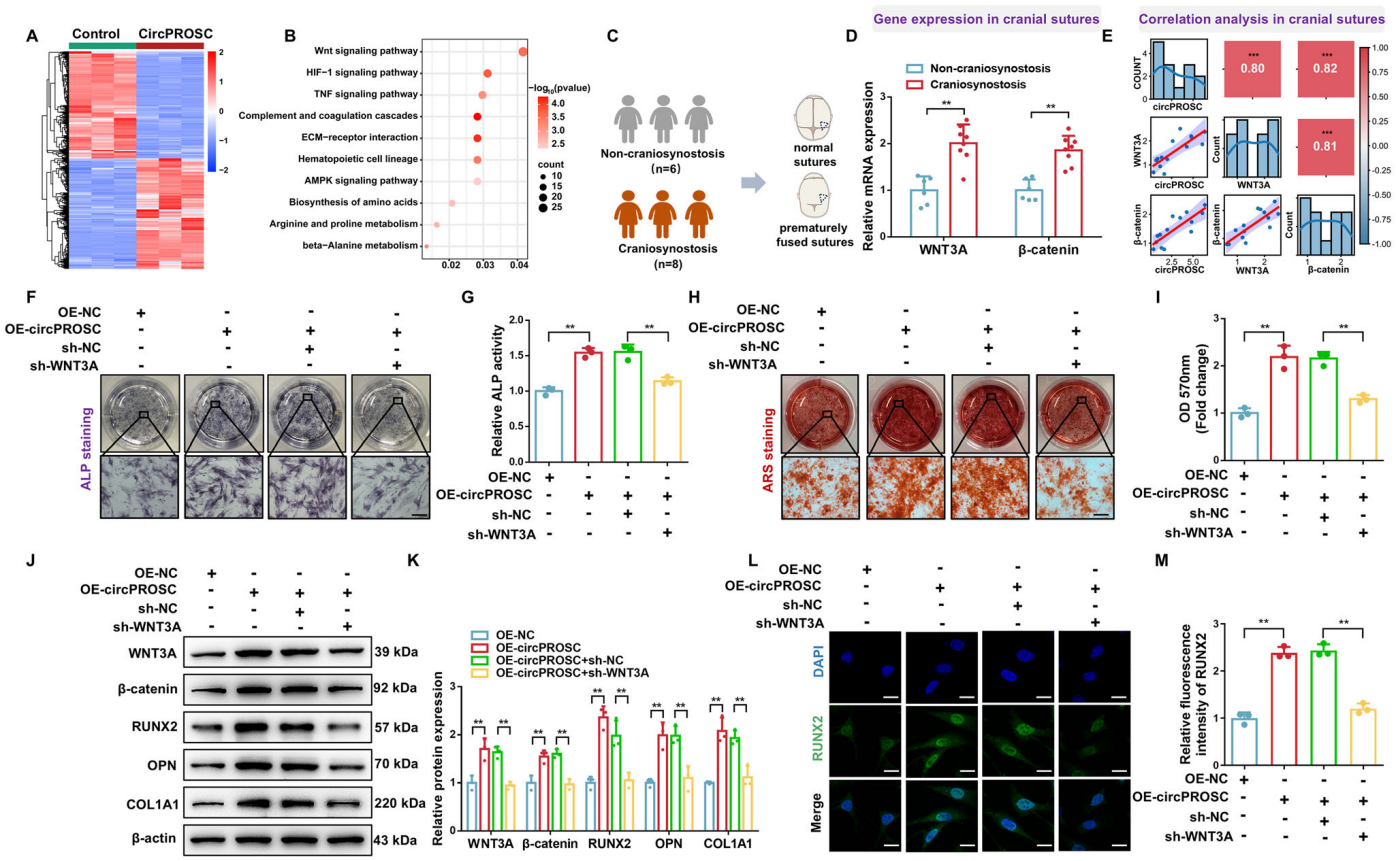
CircPROSC facilitates the osteogenic differentiation of hMSCs through regulating WNT3A. A) Heatmap displaying the differentially expressed genes (DEGs) in hMSCs with different circPROSC levels (*n*  =  3). B) KEGG enrichment analyses of DEGs after circPROSC overexpression. C) Cranial suture tissues were collected surgically from the non‐craniosynostosis group and the craniosynostosis group. D) qRT‒PCR analysis of WNT3A and β‐catenin expression in cranial sutures of non‐craniosynostosis (*n * =  6) and craniosynostosis patients (*n*  =  8). E) Correlation analysis of circPROSC with WNT3A and β‐catenin expression in cranial suture tissues. F) ALP staining of hMSCs on day 7 after osteoblast differentiation. Scale bar = 200 µm. G) ALP activity assay on day 7 after osteoblast differentiation. H) Alizarin Red staining of hMSCs on day 14 after osteoblast differentiation. Scale bar = 200 µm. I) Semiquantitative analysis of ARS staining. J) Western blot analysis and K) relative protein levels of WNT3A, β‐catenin, RUNX2, OPN, and COL1A1. L) Cellular immunofluorescence analysis of RUNX2 expression on day 7 after osteoblast differentiation. Green represents RUNX2 staining, and blue represents nuclei; Scale bar = 20 µm. M) Quantification of RUNX2 fluorescence intensity in hMSCs under different treatment conditions. Data are represented as the means±SD, *n*  =  3 independent biological replicates. Statistical significance was determined by one‐way ANOVA. *p < 0.05 and **p < 0.01.

To clarify the specific mechanism by which circPROSC regulates osteogenic effects, we overexpressed circPROSC in hMSCs and performed RNA‐seq (**Figure** **3**A). Gene Ontology (GO) analysis revealed that the differentially expressed genes were significantly enriched in pathways related to extracellular matrix organization, ossification and bone mineralization (Figure S7, Supporting Information). KEGG enrichment analysis showed that the Wnt signaling was activated by circPROSC overexpression (Figure 3B). Wnt signaling has previously been shown to be functionally related to osteogenic differentiation, where Wnt family member 3A (WNT3A), which regulates β‐catenin, is essential for the development of the skull.^[^ ^31^ ^]^ Notably, circPROSC exhibited a positive correlation with WNT3A and β‐catenin, which were highly expressed in the skulls of craniosynostosis patients, accompanied by increased nuclear β‐catenin levels and elevated expression of the downstream targets LEF1 and TCF7 (Figure 3C–E; Figure S8, Supporting Information). To demonstrate the involvement of the WNT3A/β‐catenin signaling pathway in the circPROSC‐mediated osteogenic differentiation of hMSCs, we co‐transfected hMSCs with circPROSC overexpression and WNT3A knockdown lentivirus. Functional assays elucidated that circPROSC‐mediated increases in ALP content, ALP activity and the mineralization capacity of hMSCs were largely eliminated upon WNT3A knockdown (Figure 3F–I). Overexpression of circPROSC resulted in the upregulation of WNT3A, β‐catenin, RUNX2, OPN, and COL1A1, together with increased nuclear β‐catenin levels and elevated expression of the downstream β‐catenin targets LEF1 and TCF7, whereas the downregulation of WNT3A notably counteracted the regulatory effect of circPROSC on these osteogenic markers (Figure 3J–M; Figure S9, Supporting Information). These results indicate that circPROSC promotes the osteogenic differentiation of hMSCs by functionally targeting and enhancing the expression of the WNT3A/β‐catenin signaling pathway.

### 2.4 CircPROSC Contributes to Osteogenic Differentiation by Targeting miR‐6815‐5p

**Figure 4:**
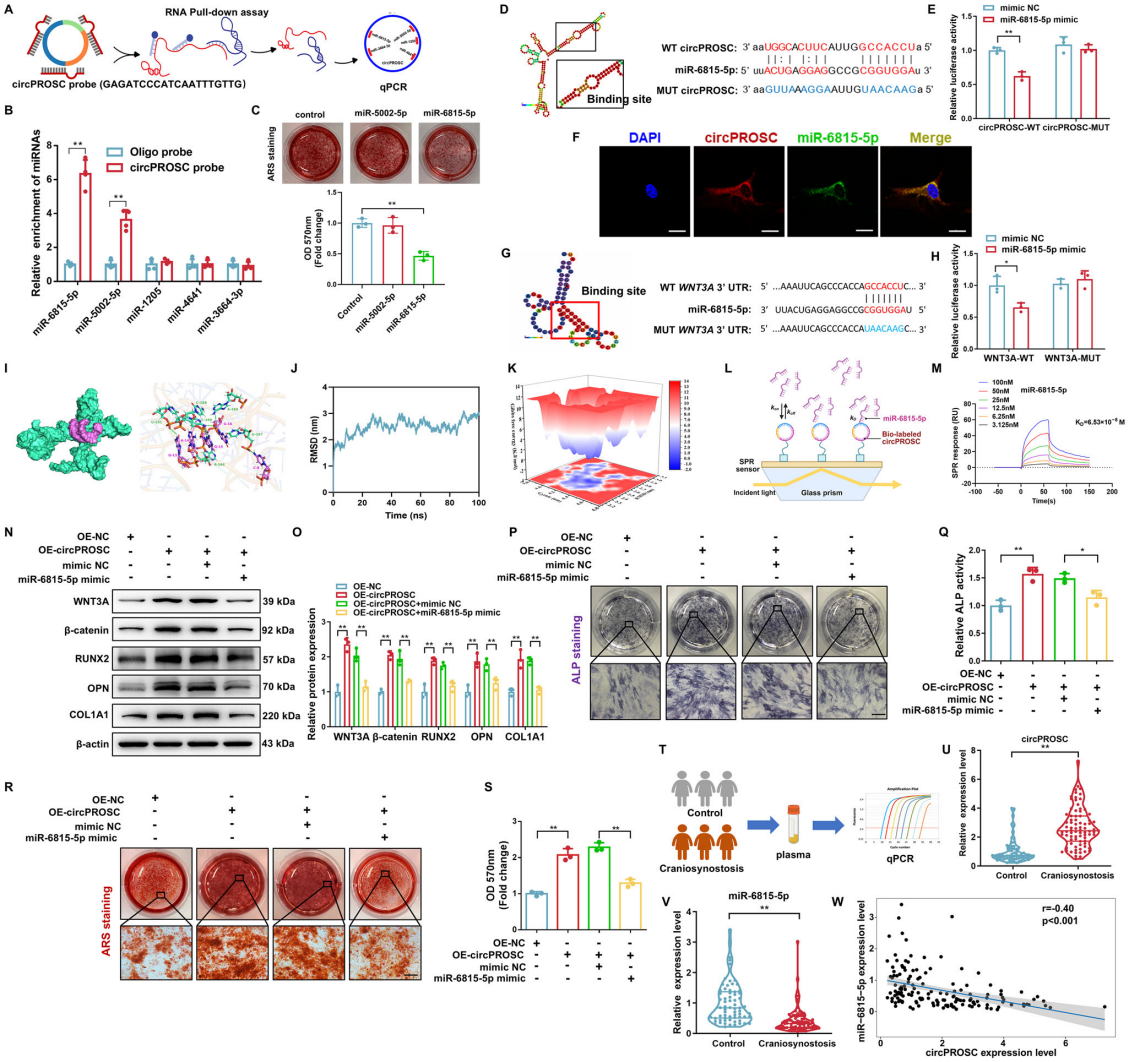
CircPROSC contributes to osteogenic differentiation by targeting miR‐6815‐5p. A) Flowchart of the RNA pulled down experiment. B) qPCR analysis of the predicted miRNAs pulled down by circPROSC. C) Alizarin red staining of hMSCs after osteogenic differentiation for 14 days following transfection. D) Predicted binding site of miR‐6815‐5p on circPROSC using RNAalifold. E) Luciferase activity of circPROSC‐WT and circPROSC‐MUT upon miR‐6815‐5p overexpression. F) Colocalization of circPROSC (red) and miR‐6815‐5p (green) in hMSCs determined by fluorescence in situ hybridization (FISH). Scale bar = 20 µm. G) Predicted binding site of miR‐6815‐5p on the 3′UTR of WNT3A using RNAalifold. H) Luciferase activity of WNT3A‐WT and WNT3A‐MUT upon miR‐6815‐5p overexpression. I) 3D surface representation showing the binding interface between circPROSC (cyan) and miR‐6815‐5p (magenta), and a detailed view of the docking model illustrating the intermolecular interactions, including hydrogen bonds and base stacking, between circPROSC nucleotides and miR‐6815‐5p. J) Root mean square deviation (RMSD) of the complex, representing global structural stability. K) 3D diagram of FEL showing the global minimum energy basin and conformational stability. L) Schematic representation of SPR analysis for the circPROSC–miR‐6815‐5p interaction. M) SPR results of the circPROSC–miR‐6815‐5p interaction. N) Western blot analysis and O) relative protein levels of WNT3A, β‐catenin, RUNX2, OPN, and COL1A1. P) ALP staining of hMSCs on day 7 after osteogenic differentiation. Scale bar = 200 µm. Q) ALP activity assay on day 7 after osteogenic differentiation. R) Alizarin Red staining of hMSCs on day 14 after osteogenic differentiation. Scale bar = 200 µm. S) Semiquantitative analysis of ARS staining. T) Diagram showing the detection of plasma miR‐6815‐5p and circPROSC levels in patients with craniosynostosis (*n * =  81) and healthy individuals (*n * =  67). U) Plasma circPROSC levels determined by qRT‐PCR. V) Plasma miR‐6815‐5p levels determined by qRT‐PCR. W) Correlation between circPROSC and miR‐6815‐5p levels in plasma. Data are represented as the means±SD, n  =  3 independent experiments. Statistical significance was determined by one‐way ANOVA. *p < 0.05 and **p < 0.01.

Molecular docking predicted that circPROSC could bind to AGO2, which was verified by RIP experiments (Figure S10, Supporting Information), indicating that circPROSC may function as a miRNA sponge. miRNA target prediction software from Arraystar based on TargetScan and miRanda, was used to predict the interactions between circRNAs and miRNAs (Figure S11, Supporting Information). Notably, among these candidate miRNAs, miR‐5002‐5p and miR‐6815‐5p were abundant in the pull‐down of the biotin‐labeled circPROSC probe pull‐down (**Figure** **4**A,B). However, we observed that only the miR‐6815‐5p mimic inhibited osteogenesis in hMSCs, as evidenced by reduced mineralization capacity, ALP content, ALP activity, and osteogenic protein levels, and this effect was dose‐dependent (Figure 4C; Figures S12 and S13, Supporting Information). qRT‐PCR analysis further confirmed that miR‐6815‐5p was expressed at physiological levels, showing progressive downregulation during hMSC osteogenic differentiation (941.95 ± 76.42 fM at Day 0, 646.69 ± 19.30 fM at Day 3, and 377.80 ± 31.68 fM at Day 7) and lower concentrations in craniosynostosis sutures compared with controls (481.74 ± 75.21 fM vs 930.59 ± 79.79 fM) (Figure S14, Supporting Information). The dual‐luciferase reporter assay demonstrated that the luciferase activity in the WT reporter was significantly reduced by the miR‐6815‐5p mimic, while the MUT reporter remained unchanged (Figure 4D,E). RNA‐FISH assays demonstrated the colocalization of circPROSC with miR‐6815‐5p in the cytoplasm (Figure 4F). Bioinformatics analysis predicted a putative binding site between miR‐6815‐5p and the 3′UTR of WNT3A, and the luciferase reporter assay further demonstrated that miR‐6815‐5p overexpression significantly quenched the WT WNT3A 3′UTR reporter while having no effect on the MUT WNT3A 3′UTR reporter. (Figure 4G,H). Additionally, molecular docking and molecular dynamics (MD) simulations revealed a highly stable interaction between circPROSC and miR‐6815‐5p (Figure 4I). The circPROSC–miR‐6815‐5p complex rapidly equilibrated, with a root mean square deviation (RMSD) stabilizing at 2.48 ± 0.32 nm, a stable radius of gyration (Rg), and low RMSF values at the binding interface. (Figure 4J; Figure S15A,B, Supporting Information). Solvent‐accessible surface area (SASA) analysis indicated a compact conformation, which was further stabilized by an average of 2.79 intermolecular hydrogen bonds (maximum 9) (Figure S15C,D, Supporting Information). Free energy landscape analysis revealed a single deep minimum, with a total binding energy of −127.3 kJ/mol (Figure 4K; Figure S15E and Table S2, Supporting Information). Consistently, surface plasmon resonance (SPR) analysis revealed dose‐dependent binding, with a K_D_ of 6.53 × 10^−8^ M, confirming high‐affinity circPROSC–miR‐6815‐5p interaction (Figure 4L,M). circPROSC knockdown significantly elevated miR‐6815‐5p levels in hMSCs, while overexpression reduced its abundance, supporting its role as a dynamic and functional miRNA sponge (Figure S16, Supporting Information). Furthermore, we conducted a rescue experiment to explore whether circPROSC promoted the osteogenic differentiation of hMSCs by interacting with miR‐6815‐5p. We found that the overexpression of circPROSC resulted in a significant increase in the levels of WNT3A, β‐catenin, nuclear β‐catenin, and the downstream β‐catenin targets LEF1 and TCF7, as well as RUNX2, OPN and COL1A1, an effect that was attenuated upon reintroduction of miR‐6815‐5p (Figure 4N,O; Figure S17, Supporting Information). As presented by ALP staining, ALP activity assay, and ARS staining, the promoting effect of circPROSC on osteogenesis of hMSCs was largely reversed by the miR‐6815‐5p mimic (Figure 4P–S). The expression level of plasma circPROSC was significantly higher in craniosynostosis patients than in healthy children, whereas miR‐6815‐5p showed an opposite trend (Figure 4T–V). As shown in Figure 4W, we observed a negative correlation between plasma circPROSC and miR‐6815‐5p (r = −0.40, *p* < 0.01). Collectively, these results demonstrate that circPROSC positively regulates osteogenesis in hMSCs through sponging miR‐6815‐5p.

### 2.5 Preparation and Characterization of MM@Lipo/siRNA

**Figure 5:**
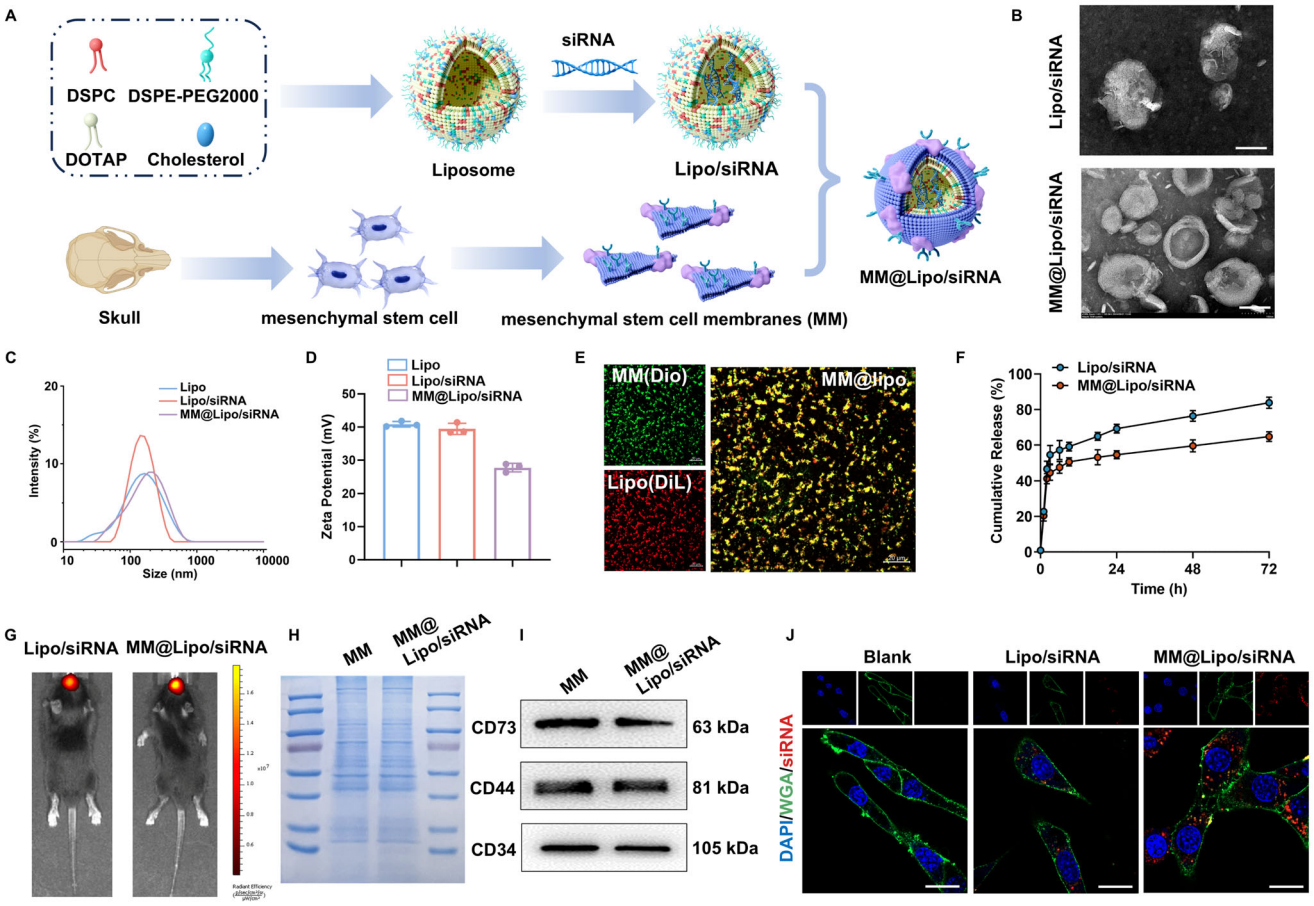
Preparation and characterization of MM@Lipo/siRNA. A) Schematic illustration of the preparation of MM@Lipo/siRNA. B) TEM images of Lipo/siRNA and MM@Lipo/siRNA (scale bar  = 100 nm). C) Hydrodynamic particle size and D) zeta potential of Lipo, Lipo/siRNA, and MM@Lipo/siRNA (*n*  =  3, means ± SD). E) Confocal fluorescence microscopy of a mixture of MM@Lipo/siRNA. Red indicates Lipo and green indicates MM. Scale bar = 20 µm. F) siRNA release from Lipo/siRNA and MM@Lipo/siRNA in PBS (pH 7.4). G) Biodistribution of the nanomedicine in the skull. H) Protein components determined by SDS‐PAGE. I) Western blot analysis of CD73, CD44, and CD34 in MM and MM@Lipo/siRNA. J) Cellular uptake of Cy5‐siRNA by cells treated with Lipo/siRNA or MM@Lipo/siRNA. Data are represented as the means±SD, *n*  =  3 independent experiments.

We next investigated the effect of circPROSC reduction on craniosynostosis in mice. To prepare biomimetic MM@Lipo/siRNA nanoparticles (**Figure** **5**A), cell membranes were isolated from mouse cranial bone MSCs and coated onto Lipo/siRNA nanoparticles by extrusion.^[^ ^32^ ^]^ TEM images revealed that both the Lipo/siRNA and MM@Lipo/siRNA nanoparticles were spherical in shape. After membrane modification, a thin membrane coating was observed onMM@Lipo/siRNA (Figure 5B). Dynamic light scattering (DLS) results showed that the average hydrodynamic diameters of Lipo, Lipo/siRNA, and MM@Lipo/siRNA were 192.40 ± 4.62, 192.60 ± 5.23, and 206.64 ± 9.70 nm, respectively (Figure 5C). Moreover, the polydispersity index (PDI) values for Lipo, Lipo/siRNA, and MM@Lipo/siRNA were 0.24 ± 0.01, 0.25 ± 0.01, and 0.29 ± 0.01, respectively (Figure S18, Supporting Information). The zeta potentials of Lipo, Lipo/siRNA, and MM@Lipo/siRNA were 40.87 ± 0.83, 39.46 ± 1.68, and 27.80 ± 1.25 mV, respectively (Figure 5D). As shown in Figure 5E, mixing DiO‐labeled MM with Dil‐labeled Lipo resulted in separate green and red fluorescence signals, and in MM@Lipo/siRNA, overlapping yellow fluorescence was observed, indicating that the MSC membrane was successfully coated on the nanoparticles. Next, we examined the release behavior of siRNAs in PBS (pH 7.4) at 37 °C. After 72 h, the release rates of MM@Lipo/siRNA and Lipo/siRNA were 64.7% and 83.8%, respectively (Figure 5F). In addition, quantitative analysis demonstrated that the relative fluorescence intensity of MM@Lipo/siRNA in the skull was significantly higher than that of Lipo/siRNA (*p* < 0.01) (Figure 5G; Figure S19, Supporting Information), whereas no detectable fluorescence signal was observed in the heart, liver, spleen, lungs, kidneys, or brain (Figure S20, Supporting Information). The protein components in MM@Lipo/siRNA nanomedicine showed a distribution similar to the native MSC membrane, confirming that membrane proteins were preserved during MM@Lipo/siRNA preparation (Figure 5H). Western blot analysis confirmed that MM@Lipo/siRNA retained the MM‐specific markers CD73, CD44, and CD34, further confirming the retention of MSC membrane proteins and their biological activity (Figure 5I). Quantitative analysis further demonstrated that the retention ratios of CD73, CD44, and CD34 between MM@Lipo/siRNA and native membranes were 0.91±0.03, 0.78±0.05, and 0.83±0.04, respectively, corresponding to an average coating efficiency of 83.9%±6.5%, thereby confirming effective membrane integration (Figure S21, Supporting Information). Since nanoparticle/gene complexes are considered to be rapidly cleared by macrophages upon in vivo delivery, we investigated whether cell membrane‐coated nanoparticles could reduce this clearance effect. After MM@Lipo/siRNA complexes were incubated with RAW264.7 cells, less siRNA was detected in the RAW264.7 cells, suggesting that the cell membrane coating of Lipo/siRNA complexes enhances biocompatibility (Figure S22, Supporting Information). Finally, the siRNA transfection efficiency of the MM@Lipo/siRNA complex was investigated in cranial suture MSCs, and the siRNA signal was found to be increased in the MM@Lipo/siRNA group compared with the Lipo/siRNA group (Figure 5J).

### 2.6 Reduction in circPROSC Inhibits Premature Fusion of the Coronal Suture of the Skull in Mice with Craniosynostosis

**Figure 6:**
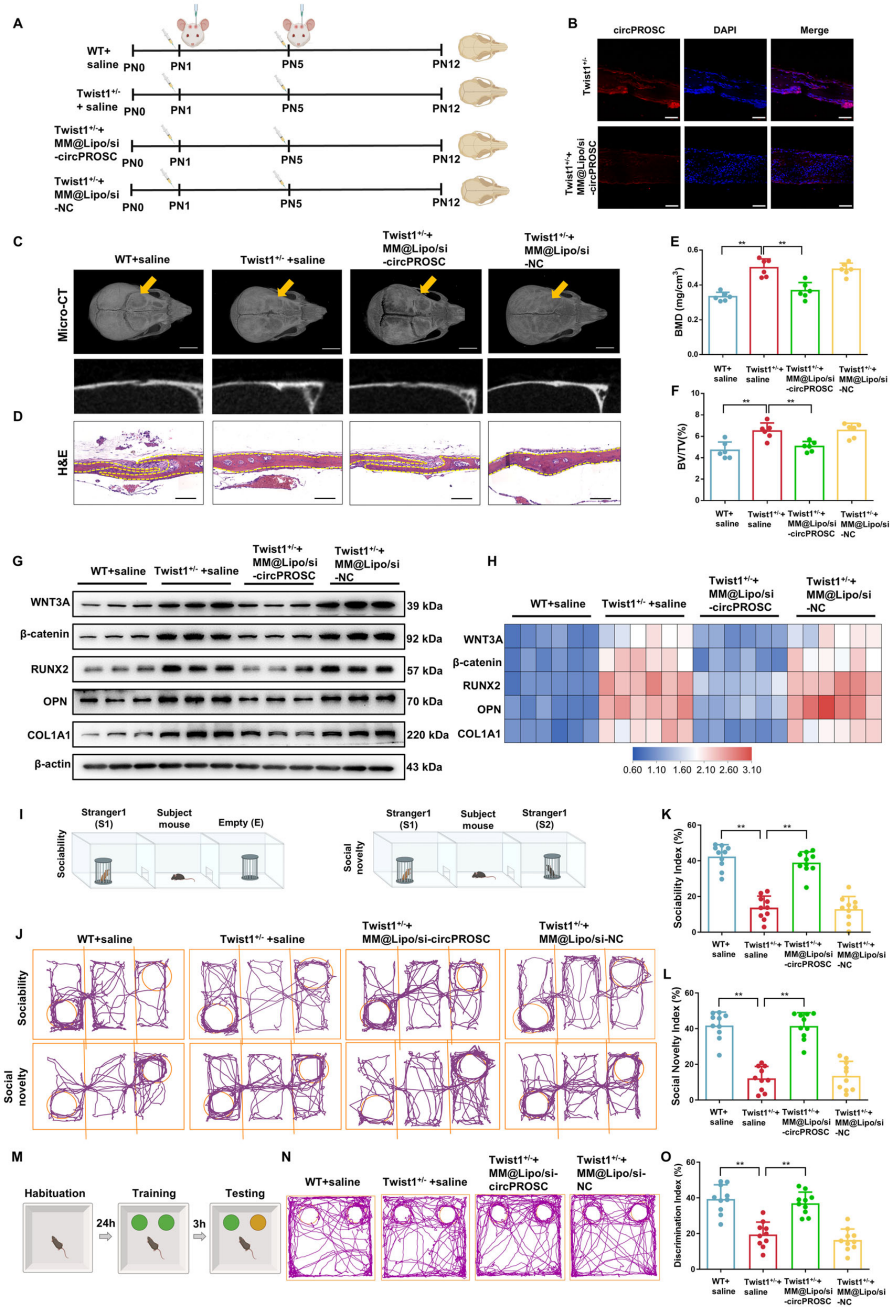
Reduction in circPROSC inhibits premature fusion of the coronal suture of the skull in mice with craniosynostosis. A) Experimental timeline and treatment schematic for Twist1^+/−^ mice. B) circPROSC levels in the coronal suture of mice (*n*  =  6). C) Representative micro‐CT images (upper panel, 3D reconstruction; lower panel, slice) of the mouse skull. Scale bar = 1 mm; the yellow arrow indicates the coronal suture (*n*  =  6). D) Representative hematoxylin and eosin (H&E) staining of the coronal suture sections from mouse skull (*n*  =  6). Scale bar = 100 µm. E) Quantitative analyses of BMD and BV/TV F) of coronal suture tissues (*n* = 6). G) Western blot analysis and H) the relative protein levels of WNT3A, β‐catenin, RUNX2, OPN, and COL1A1. *n * =  6 biologically independent mice. Statistical significance was determined by one‐way ANOVA followed by Tukey's post hoc test. I) Schematics of the three‐chamber test. Representative animal tracks J), sociability indices K), and social novelty indices L) in the three‐chamber test (*n*  =  10). M) Schematics of the novel object recognition test. Representative animal tracks N) and discrimination index O) in the novel object recognition test (*n*  =  10). Data are represented as the means±SD. Statistical significance was determined by one‐way ANOVA. *p < 0.05 and **p < 0.01.

Based on the above results, an in vivo study was performed to evaluate the therapeutic efficacy of MM@Lipo/siRNA in Twist1^+/−^ craniosynostotic mice. Each group consisted of six biologically independent mice. RT‐qPCR analysis showed that circPROSC expression was significantly upregulated in the fused coronal sutures of Twist1^+/–^ mice compared to wild‐type controls (Figures S23 and S24, Supporting Information), confirming the pathological relevance of circPROSC in this model. The study revealed that MM@Lipo/siRNA diminished circPROSC expression in mice (**Figure** **6**A,B). Micro‐CT results demonstrated that treatment with MM@Lipo/siRNA significantly mitigated the premature closure of the coronal suture in Twist1^+/−^ mice compared to negative control (NC) treatment (Figure 6C). Histological analysis showed that si‐circPROSC treatment resulted in a reduction of the overlapping area of osteogenic fronts within the coronal sutures of Twist1^+/−^ mouse skulls (Figure 6D). Furthermore, si‐circPROSC efficiently attenuated the significant increase in static bone histomorphometric indices (BMD and BV/TV) observed in Twist1^+/−^ mice (Figure 6E,F). To further assess the consistency of therapeutic response, we calculated the coefficients of variation (CVs) for BMD and BV/TV in the MM@Lipo/si‐circPROSC treatment group, which were 12.7% for BMD and 9.2% for BV/TV, indicating relatively uniform phenotypic outcomes. At the molecular level, we observed significant upregulation of WNT3A, β‐catenin and osteogenic markers in the coronal suture of Twist1^+/−^ mice, with a concomitant increase in nuclear β‐catenin levels and the downstream β‐catenin targets LEF1 and TCF7, which was largely rescued by si‐circPROSC treatment (Figure 6G,H; Figure S25, Supporting Information). A series of behavioral assessments were conducted to evaluate functional outcomes following treatment. In the three‐chamber social interaction test, circPROSC knockdown partially restored social behaviors in Twist1^+/−^ mice (Figure 6I–L). Similarly, the novel object recognition test showed that si‐circPROSC administration rescued the impaired preference for novel objects observed in Twist1^+/−^ mice (Figure 6M–O). No significant improvement in motor learning ability was observed after si‐circPROSC treatment in the rotarod test (Figure S26, Supporting Information). These data support the concept that, in craniosynostotic mice, circPROSC knockdown contributes to preventing premature fusion of cranial sutures.

### 2.7 Biosafety Assessment

Histopathological examination by H&E staining of major organs (heart, liver, spleen, lung, kidney, and brain) at postnatal day 21 (PN21) revealed no pathological abnormalities or tissue damage in any treatment group, supporting the long‐term safety of MM@Lipo/si‐circPROSC (Figure S27A, Supporting Information). To further assess immunogenicity, major organs were examined for macrophage infiltration and pro‐inflammatory cytokine expression. Immunohistochemical staining of F4/80 (a macrophage marker) and cytokine analysis of IL‐1β showed no significant increases in the MM@Lipo/si‐circPROSC group, indicating low systemic immunogenicity (Figure S27B,C, Supporting Information). Collectively, these findings confirm the favorable biosafety profile of MM@Lipo/si‐circPROSC nanoparticles for in vivo application.

## 3 Discussion

The lack of a molecular target poses a significant challenge for the early diagnosis and treatment of craniosynostosis. In this study, we identified a novel predictive biomarker, circPROSC, which was found to be overexpressed in both the plasma and cranial sutures of patients with craniosynostosis. Mechanistically, circPROSC promoted the osteogenic differentiation of MSCs through miR‐6815‐5p‐mediated modulation of the Wnt signaling pathway. In addition, the therapeutic potential of circPROSC was demonstrated for the first time through microinjection of MM@Lipo/si‐circPROSC into a craniosynostosis mouse model.

In recent years, research on circRNA in the diagnosis and treatment of bone diseases has made rapid progress.^[^ ^16^, ^33^ ^]^ Studies have found that specific circRNAs are differentially expressed in osteoblasts and osteoclasts, potentially regulating bone metabolism and participating in disease progression.^[^ ^34^, ^35^ ^]^ Based on evidence from circRNA expression analysis, circPROSC is highly expressed in the cranial suture tissues of patients with craniosynostosis and is positively correlated with the degree of osteogenic differentiation of MSCs. These observations suggest that circPROSC may be a craniosynostosis‐specific circRNA. Notably, plasma circPROSC levels are significantly higher in craniosynostosis patients than in healthy individuals. Furthermore, circPROSC exhibited excellent stability in serum, remained stable at room temperature, and was resistant to multiple freeze‐thaw cycles, indicating its potential as a diagnostic biomarker. In terms of treatment, knockdown of circPROSC significantly inhibited osteogenic differentiation in both the nude mouse ectopic bone formation model and the craniosynostosis mouse model, highlighting its potential as a therapeutic target. In many cases of craniosynostosis, post‐surgical resynostosis occurs, necessitating re‐operation.^[^ ^36^ ^]^ Current preventive measures, such as orthopedic helmets, fail to precisely regulate local bone metabolism. Based on this, we propose using engineered MSCs to target and deliver si‐circPROSC molecules directly to the cranial suture area, inhibiting osteogenic differentiation to prevent the formation of bony bridges, while also ameliorating neurobehavioral deficits such as impaired social interaction and cognition associated with craniosynostosis. The advantage of this strategy lies in its high specificity and targeting capability. The findings of this study provide important insights for the development of minimally invasive therapeutic strategies based on circPROSC. These findings underscore that the pathogenesis of craniosynostosis involves not only excessive bone formation but also disruption of the cranial suture microenvironment. MSCs residing in the suture mesenchyme are essential for maintaining suture patency during skull growth.^[^ ^37^ ^]^ CircPROSC exerts its pathological function primarily by dysregulating the fate of these MSCs, promoting their premature osteogenic differentiation. Importantly, inhibition of circPROSC in vivo significantly restored suture openness and fibrous tissue integrity, suggesting that therapeutic targeting of circPROSC may re‐establish normal suture biology.

RNA‐seq of circPROSC‐overexpression MSCs revealed that the Wnt signaling pathway is one of the primary pathways targeted by circPROSC overexpression in MSCs. The Wnt pathway is closely associated with craniofacial development during early embryogenesis, with β‐catenin serving as a core molecule that regulates the transcription of numerous genes within the Wnt signaling cascade.^[^ ^38^ ^]^ At the molecular level, haploinsufficiency of Twist1 leads to impaired Axin2 expression and enhanced Wnt signaling, contributing to craniosynostosis.^[^ ^39^ ^]^ By implanting exogenous MSCs, the activity of the Wnt pathway can be modulated, restoring it to normal levels and preventing postoperative resynostosis, supporting that the Wnt signaling pathway could be a crucial therapeutic target for craniosynostosis. Previous studies have shown that the competing endogenous RNA (ceRNA) network plays a significant role in human diseases, including osteogenic differentiation.^[^ ^40^, ^41^ ^]^ Consistent with these findings, we found that circPROSC predominantly localizes to the cytoplasm of MSCs and has complementary sequences with the seed region of miR‐6815‐5p. Luciferase reporter assays, FISH, and RAP analyses consistently demonstrated that circPROSC directly binds to miR‐6815‐5p. In addition, WNT3A is a target of miR‐6815‐5p, and the elevated expression of WNT3A and β‐catenin induced by circPROSC overexpression was rescued by miR‐6815‐5p mimics. Therefore, circPROSC regulates the osteogenic differentiation of MSCs through the miR‐6815‐5p/Wnt/β‐catenin axis.

The molecular pathogenesis of craniosynostosis is typically attributed to the overactivation of pro‐osteogenic signaling cascades. For example, gain‐of‐function mutations in FGFR genes lead to sustained activation of downstream MAPK/ERK signaling, thereby inducing premature suture fusion.^[^ ^42^ ^]^ Similarly, aberrant BMP signaling, usually mediated by SMAD protein phosphorylation, activates RUNX2 and induces premature osteogenesis of the suture mesenchyme.^[^ ^43^ ^]^ Notably, our study introduces a regulatory layer at the non‐coding RNA level: circPROSC binds to and inhibits miR‐6815‐5p, thereby relieving its suppression of WNT3A and activating the canonical Wnt/β‐catenin signaling pathway. Both the classical FGFR/BMP signaling pathways and the newly defined circPROSC/Wnt axis converge on RUNX2‐mediated osteogenic gene expression, but the circPROSC pathway adds an epigenetic regulatory dimension. This is particularly important for the majority of craniosynostosis patients who lack identifiable coding mutations. Therefore, our data suggest that circPROSC‐driven Wnt activation may function independently of, or synergistically with, FGFR/BMP pathway perturbations, thereby expanding the mechanistic landscape of cranial suture biology and providing alternative molecular targets for intervention.

Currently, the only treatment for craniosynostosis is correction of the skull deformity through complex surgery.^[^ ^44^ ^]^ However, these procedures are invasive and associated with the risks of potential complications, highlighting the urgent need for minimally invasive treatments. Recently, the FDA approved a phase 3 clinical trial of patisiran, an RNAi‐based therapy for transthyretin amyloidosis (ATTR)^[^ ^45^ ^]^ Additionally, the construction of artificial loops containing miRNA or protein binding sites using enzyme‐linked methods can be employed to specifically target the dysfunction of miRNAs or proteins implicated in disease progression.^[^ ^46^, ^47^ ^]^ Despite these advances, RNA‐based therapies still face challenges such as limited targeted delivery, instability, and susceptibility to degradation. To protect circPROSC siRNA from hydrolysis while enabling immune evasion and cell‐specific recognition, we developed MSC membrane‐coated nanoparticles loaded with circPROSC siRNA. These nanoparticles exhibit excellent biocompatibility, enhanced cell targeting, and uptake efficiency, enabling effective delivery of siRNA to cranial sutures, thus improving the symptoms of cranial suture fusion in a mouse model of craniosynostosis. These findings provide a strategy for the minimally invasive treatment of craniosynostosis, with potential implications for clinical practice. Although our samples were all derived from non‐syndromic craniosynostosis (NSC) patients to minimize genetic heterogeneity, we used the widely recognized Twist1^+/−^ mouse model for in vivo validation. While the Twist1^+/−^ mouse is a syndromic model, it exhibits isolated coronal suture fusion and MSC‐mediated osteogenic dysregulation, which, histologically, resembles the pathological features of NSC.^[^ ^48^ ^]^ Moreover, our therapeutic strategy targets downstream pathways regulated by circPROSC, such as the Wnt signaling pathway and MSC differentiation, which are present in both syndromic and NSC. Therefore, the observed rescue effect in this model reflects the shared pathological mechanisms between syndromic and NSC, supporting the broader applicability of our findings. However, it should be noted that, to date, a mature NSC mouse model has yet to be established. Therefore, this study used the Twist1^+/−^ mouse model, which is the closest available model to NSC, but this may limit its ability to accurately replicate the heterogeneous etiologies observed in clinical cases. Future research utilizing emerging or newly developed non‐syndromic models will be crucial for rigorously validating the therapeutic efficacy of MM@Lipo/si‐circPROSC, better defining its translational applicability, and ensuring its broader clinical relevance.

In this study, we investigated the mechanism underlying the regulation of craniosynostosis by circPROSC and identified it as a pivotal regulator of osteogenic differentiation. Through the microinjection of MM@Lipo/si‐circPROSC to interfere with circPROSC expression, we aimed to avoid surgery and alleviate premature closure of cranial sutures. However, it is crucial to emphasize that the elevated expression of circPROSC in craniosynostosis most likely represents a pathogenic driver rather than a compensatory response. Therefore, our proposed therapeutic approach focuses on inhibiting circPROSC overactivation to suppress excessive osteogenic differentiation. Although preliminary results in the Twist1^+/–^ mouse model support the pathogenic mechanism and suggest potential therapeutic value, further investigations using additional in vivo models and more comprehensive functional assessments, including intracranial pressure measurement, are needed to clarify its clinical translational potential. In addition, the function and applicability of circPROSC in various craniosynostosis models require further evaluation and investigation. Moreover, several challenges remain for clinical implementation, including the need to define the optimal therapeutic window during infancy, evaluate long‐term safety and immunogenicity, and establish scalable and standardized production of MSC‐membrane nanoparticles. In future studies, elucidating how circPROSC functions within complex biological cascades in vivo, especially in human systems, will be crucial for promoting clinical translation.

## 4 Conclusion

Our findings highlight circPROSC as a novel circRNA associated with craniosynostosis that holds promise as an early diagnostic tool and therapeutic target. MSC membrane‐coated nanoparticles loaded with circPROSC siRNA have the advantages of non‐invasiveness and high biocompatibility. Our data provide additional evidence for the role of circPROSC in the pathogenesis of craniosynostosis, and the therapy based on circPROSC siRNA specific to cranial sutures may hold significant potential for clinical application in the treatment of craniosynostosis.

## 5 Experimental Section

### Study Subjects and Clinical Samples

Initially, 136 craniosynostosis patients and 102 age‐matched healthy children (2–3 years old) without a history of prior craniofacial surgery were screened for eligibility at the Children's Hospital Affiliated to Nanjing Medical University. For the craniosynostosis group, inclusion criteria were as follows: a) imaging‐confirmed craniosynostosis and age 2–3 years. Exclusion criteria included: a) patients diagnosed with syndromic craniosynostosis; b) confirmed pathogenic variants in genes commonly implicated in craniosynostosis, such as TWIST1/2, ephrin‐B1 (EFNB1), fibroblast growth factor receptors (FGFR1/2/3), and muscle segment homeobox 2 (MSX2); c) chromosomal abnormalities; d) maternal use of known teratogenic medications during pregnancy (e.g., isotretinoin, valproic acid, phenytoin, methotrexate); and e) patients who refused to provide blood samples or had insufficient clinical data. For the control group, exclusion criteria included: a) abnormal cranial bone structures or brain anomalies identified by imaging; b) history of neurodevelopmental delay, intellectual disability, or other congenital developmental disorders; and c) refusal to provide blood samples or insufficient clinical data. After applying these criteria, 81 non‐syndromic craniosynostosis (NSC) patients and 67 healthy controls were included in the final analysis. The sample selection process is illustrated in Figure S1 (Supporting Information). The research protocol was approved by the Ethics Committee of the Children's Hospital Affiliated to Nanjing Medical University (Approval No. 202312004‐1). The general characteristics of the study population are shown in Table S1 (Supporting Information).

### Animals

BALB/c nude mice (6 weeks old, female) and C57BL/6J wild‐type (WT) mice (8 weeks old, female) were obtained from the Experimental Animal Center of Nanjing Medical University. Heterozygous Twist1 knockout (Twist1^+/−^) mice (8 weeks old, male) were purchased from GemPharmatech Co., Ltd. These animals were housed under specific pathogen‐free (SPF) conditions with an ambient temperature of 22±2 °C, a relative humidity of 30%–70%, and a 12 h light/dark cycle, with free access to standard chow and water. Only Twist1^+/−^ mice were subsequently bred in‐house, and genotyping was performed for each generation to confirm heterozygosity before inclusion in experiments. Genomic DNA was extracted from tail biopsies collected at postnatal day 1 (PN1) using the One Step Mouse Genotyping Kit (Vazyme Biotech, China) according to the manufacturer's instructions. Two primer pairs were used for PCR amplification: Primer 1 forward 5′‐ATCCCTAAACCCTGCTGCATGG‐3′ and reverse 5′‐CCTGGAGATGCAGGCTTATCTAAGTG‐3′; Primer 2 forward 5′‐AGACCAAATTCACAAGAATCAGGGC‐3′ and reverse 5′‐TGATTTTGCAGGCCAGTTTGATC‐3′. PCR was performed using 2× Taq Plus Master Mix (Dye Plus, Vazyme Biotech, China) with the following conditions: 94 °C for 5 min; 35 cycles of 94 °C for 30 s, 55 °C for 30 s, and 72 °C for 30 s; final extension at 72 °C for 5 min. PCR products were separated on a 2% agarose gel, stained with GelRed (Biotium, Hayward, CA, USA), and visualized using a Gene Genius Bio‐Imaging System (Syngene, Cambridge, UK). Heterozygous Twist1⁺/− mice were identified by the presence of both a 289 bp fragment (Primer 1) and a 339 bp fragment (Primer 2), whereas Twist1⁺/⁺ mice displayed only the 339  bp fragment (Primer 2). All the animal experiments were conducted with the approval of the Institutional Animal Care and Use Committee (IACUC) of Nanjing Medical University (Approval No. IACUC‐ 2 306 021).

### Cell Culture and Osteogenic Induction

Primary human mesenchymal stem cells (hMSCs) were obtained from Cyagen Biosciences Inc., Suzhou, China, and maintained in Modified Eagle Medium‐Alpha (α‐MEM, Corning Cellgro, USA) containing 10% fetal bovine serum (HyClone) and 1% penicillin‒streptomycin (Gibco, USA) in a humidified incubator with 5% CO_2_ at 37 °C. To maintain their potential for self‐renewal and osteogenesis, hMSCs between passages 3 and 5 were used for experiments in the following study. To induce osteogenesis, hMSCs were cultured in osteogenic medium (Cyagen Biosciences Inc., USA) that contained 100 µM ascorbic acid, 2 mM β‐glycerophosphate, and 10 nM dexamethasone (Sigma‒Aldrich, USA). The osteogenesis medium was refreshed every 3 days.

### Statistical Analyses

Differences between the control and patient groups were analyzed using Student's t‐test for continuous variables or Pearson's χ^2^ test for categorical variables. The diagnostic performance of circPROSC was evaluated using receiver operating characteristic (ROC) analysis and the area under the curve (AUC). Correlation between circPROSC and osteogenic gene markers in cranial suture tissues was determined using Spearman's correlation analysis. Logistic regression was applied to assess the association between circPROSC expression and the risk of craniosynostosis. For normally distributed data, two‐group comparisons were conducted using Student's t‐test, and comparisons among more than two groups were performed using one‐way ANOVA followed by Tukey's post hoc test. For non‐normally distributed data, Mann–Whitney U test or Kruskal–Wallis test with Dunn's post hoc test was applied. All experiments were performed in at least three independent replicates, and the data are presented as the means±standard deviation (SD). Statistical analyses were performed using GraphPad Prism version 10.0 (GraphPad Software, USA), and a two‐sided *p* < 0.05 was considered statistically significant.

## Conflict of Interest

The authors declare no conflict of interest.

## Author Contributions

Z.W., X.C., J.X., and Q.Y. contributed equally to this study. Z.W. completed most of the experiments, wrote and revised the manuscript. Q.Y. provided clinical samples. X.C., J.J., and J.X. performed the statistical analyses. Q.L. performed the RNA‐seq. A.G. conceived and led the design of the study and revised the manuscript. All the authors have read and approved the final article.

## Supporting information



Supporting Information

## Data Availability

The data that support the findings of this study are available from the corresponding author upon reasonable request.
