# Supplementary material for: MSC Membrane‐Coated circPROSC‐siRNA Nanoparticles for Ameliorating Craniosynostosis by Inhibiting Premature Suture Ossification
Source: Adv Sci (Weinh). 2025 Nov 3;13(3):e10454. doi: 10.1002/advs.202510454 (PMC12806500; doi:10.1002/advs.202510454)
Supplement: Supplementary file 1 — Supporting Information [file ADVS-13-e10454-s001.pdf]

MSC Membrane-Coated circPROSC-siRNA Nanoparticles for Ameliorating Craniosynostosis by Inhibiting Premature Suture Ossification

Zhenkun Weng[1,2*], Xiu Chen[1,2*], Jin Xu[1,2*], Qing Yan[4*], Jian Jiao[1,2], Qian Liu[1,2], Aihua Gu[1,2#]

[1]State Key Laboratory of Reproductive Medicine and Offspring Health, School of Public Health, Nanjing Medical University, Nanjing, 211166, China.

[2]Jiangsu Environmental Health Risk Assessment Engineering Research Center, Key Laboratory of Modern Toxicology of Ministry of Education, Center for Global Health, Nanjing Medical University, Nanjing, 211166, China.

[3]Department of Neurosurgery, Children's Hospital of Nanjing Medical University, Nanjing, 211166, China.

*These authors contributed equally to the present study and should be regarded as joint first authors.

[#]Correspondence to:

Aihua Gu, School of Public Health, Nanjing Medical University, 101 Longmian Avenue, Nanjing 211166, China. E-mail: aihuagu@njmu.edu.cn.

Supplementary Materials and Methods

Interviews and sample collection

A structured questionnaire was used to record information including the highest educational attainment within the participants' families (junior high school and below, high school, post-secondary education and above), maternal parity, household exposure to passive smoking (yes or no), maternal age, and gestational week of delivery. None of the mothers in the study reported active smoking during pregnancy. These variables were treated as covariates in the statistical analysis conducted for the population studies. After informed consent of the guardian, fasting venous blood samples were collected, followed by separation of plasma components. Cranial suture tissues from craniosynostosis patients were obtained from resected specimens during cranial vault remodeling or suturectomy, with precise sampling from the anatomical location of the original cranial suture, that had been replaced by a bony bridge. The anatomical location of the original suture was clearly identified using preoperative CT imaging and confirmed intraoperatively with anatomical landmarks to ensure accurate sampling of the fused suture region. Control cranial suture tissues were obtained from discarded tissues of individuals with severe craniocerebral trauma who had undergone decompressive craniotomy, with precise sampling from the anatomical region corresponding to normal cranial sutures. All specimens were stored in ultra-low temperature freezers at -80 °C.

Microarray analysis (circRNA microarray)

Cranial suture tissues from craniosynostosis patients (n = 5) and healthy controls (n = 5) were first used to construct the human circRNA microarray and to identify and screen differentially expressed circRNAs (DECs). Total RNA was extracted using

TRIzol reagent (Invitrogen, USA) according to the manufacturer's instructions. To enrich circular RNAs, total RNA was digested with RNase R (Lucigen, formerly Epicentre; Cat. No. RNR07250) at 3 U/μg in RNase R buffer at 37°C for 30 min to degrade linear RNAs. Ribosomal RNAs were depleted using the RiboMinus™ Eukaryote Kit v2 (Thermo Fisher Scientific, Cat. No. A15026), and depletion efficiency was assessed with an Agilent 2100 Bioanalyzer (Agilent Technologies, Cat. No. G2939BA). The enriched circRNAs were then amplified and transcribed into fluorescent cRNA using the Arraystar Super RNA Labelling Kit (Arraystar, USA). Labelled cRNAs were hybridized onto an Arraystar Human circRNA Array (Arraystar Inc., MD, USA; Cat. No. 8x15K V2.0). Hybridization was carried out at 65°C for 17 hours in an Agilent hybridization oven (Agilent Technologies; Cat. No. G2545A). After washing the slides, the arrays were scanned using an Agilent Scanner G2505C and the results were analyzed with Agilent Feature Extraction software (version 11.0.1.1). This microarray analysis was performed by Kangcheng Biotech (Shanghai, China). Data for the circRNA microarray were analyzed using R software (version 4.2.2) and the limma package. P values were adjusted with the Benjamini‑Hochberg method to control the false discovery rate (FDR). Threshold criteria were set as $|\log_2(\text{FoldChange (FC)})| \geqslant 1$ and FDR < 0.05. Statistical significance was estimated by Student's t-test.

mRNA transcriptome sequencing

For whole transcriptome sequencing, hMSCs were collected from the circPROSC overexpression and control groups. Total RNA was extracted using TRIzol reagent (Invitrogen, USA) according to the manufacturer's instructions. Sequencing libraries were prepared using the NEBNext® Ultra™ II Directional RNA Library Prep Kit for

Illumina® (New England Biolabs, Cat. No. E7760L) following the manufacturer's protocol. Briefly, poly (A)+RNA was enriched, fragmented, and converted into cDNA. The second-strand synthesis incorporated dUTP to maintain strand specificity. After end repair, adaptor ligation, and PCR amplification (12–15 cycles), the libraries were size-selected to 250–300 bp using AMPure XP beads (Beckman Coulter, Cat. No. A63881). The qualified libraries were sequenced on the Illumina NovaSeq 6000 platform (Illumina, USA; Cat. No. 20012850) to generate 150 bp paired-end reads, yielding approximately 20 million reads per sample. Data processing and bioinformatic analyses were performed by LC-Bio Technologies (Hangzhou, China). Differentially expressed mRNAs were identified using the criteria $|log2FC| \geqslant 1$ and $P < 0.05$.

Cell transfection

sh-circPROSC, circPROSC overexpression (OE-circPROSC), OE-WNT3A, sh-WNT3A, miR-6815-5p mimic, miR-6815-5p inhibitor and corresponding negative controls (NC) were designed and synthesized by RiboBio (Guangzhou, China). The hMSCs were seeded into 6-well plates to reach approximately 50% confluence and then transfected with lentiviruses at an MOI of 50 within 12 h according to the manufacturer's manual. All target sequences were shown in Table S3.

Stability determination of circPROSC

To assess the stability of circPROSC, hMSCs were incubated with 1 U/μg of RNase R (Geneseed, R0301) for 10 min at 37 °C. To determine the stability of plasma circPROSC, plasma samples from subjects (healthy control individuals and craniosynostosis patients) were subjected to rigorous conditions, including incubation

at room temperature for 0 h, 4 h, 8 h, and 24 h, as well as multiple cycles of freezing and thawing (0, 2, 4, and 8 cycles) between -80 °C and room temperature.

Nuclear and cytoplasmic extraction

Cytoplasmic and nuclear fractions were isolated using PARIS™ Kits (Thermo Fisher Scientific, AM1556) following the manufacturer's instructions. In brief, hMSCs were lysed in Cell Fraction Buffer and incubated on ice for 30 min. Subsequently, the lysate was centrifuged at 500 × g for 3 minutes at 4 °C, and the resulting supernatant was collected as the cytoplasmic fraction. The pellet was then washed with Cell Fraction Buffer to collect the nuclei fraction.

Alkaline phosphatase (ALP) and Alizarin Red S (ARS) staining

After 7 days of osteogenic induction, the cells were fixed with 4% paraformaldehyde for 20 min at room temperature, and a BCIP/NBT staining kit (Beyotime Biotechnology, China) was performed for ALP staining. ALP activity was determined using a commercial kit according to the manufacturer’s guidelines (Jiancheng, Nanjing, China). Treated hMSCs were induced to undergo osteogenesis for 14 days and cells were fixed with 4% paraformaldehyde for 20 min, followed by incubation with 2% ARS (Sigma–Aldrich, USA) for 10 min. Semiquantitative analysis of ARS was performed by dissolving in 1 mL 10% cetylpyridinium chloride (Sigma–Aldrich USA) at 37 °C for 15 min, and the absorbance values were measured at 560 nm. Images were captured by microscope (Olympus, Japan).

Protein extraction and Western blotting analysis

RIPA lysis buffer mixed with protease inhibitor was used for total protein from cells.

The protein was isolated, and the concentration was determined with BCA kits (Beyotime, China). Equal amounts of protein samples (60 μg) were electrophoresed with SDS–PAGE and transferred to PVDF membranes (Millipore, USA). Subsequently, the membranes were blocked for 2 h with 5% nonfat milk before incubation overnight at 4 °C with primary antibodies against RUNX2 (1:1000, 12556, CST), OPN (1:1000, 22952-1-AP, Proteintech), WNT3A (1:1000, ab219412, Abcam), β-catenin (1:1000, 8480, CST), CD73 (1:1000, 12231-1-AP, Proteintech), CD44 (1:1000, 60224-1-Ig, Proteintech), CD34 (1:1000, 0108-2, HuaAn Biotechnology), LEF1 (1:1000, 2230, CST), TCF7 (1:1000, 2203, CST) and Lamin B1 (1:1000, 13435, CST). Afterward, the membranes were incubated with the corresponding secondary antibody at room temperature for 1 hour. The immune complexes were exposed using enhanced chemiluminescence (CST, USA). Band intensities were quantified with ImageJ software.

Total RNA extraction and qRT–PCR

For cranial suture tissues, approximately 50 mg of tissue was homogenized in 1 mL TRIzol reagent (Invitrogen, Cat. No.: 15596026) using a bead mill, incubated at room temperature for 5 min, followed by phase separation with chloroform and RNA precipitation using isopropanol. RNA pellets were washed with 75% ethanol and dissolved in RNase-free water. For plasma samples, total RNA was isolated from 1 mL plasma using the mirVana™ PARIS™ Kit (Thermo Fisher Scientific. Cat. No.: AM1556) as previously reported[1]. Genomic DNA contamination was eliminated using the DNA-free™ DNase Removal Kit (Thermo Fisher Scientific. Cat. No.: AM1906). RNA was subjected to reverse transcription using the PrimeScript RT reagent Kit (TaKaRa, Otsu, Japan) or miRNA 1st Strand cDNA Synthesis Kit

(Vazyme, Nanjing, China) for miRNAs. qRT–PCR was conducted with SYBR Green assays (Applied Biological Materials Inc., Canada) using a Roche LightCycler 480 II machine. The details of the primer sequences are listed in Table S4. The value of gene expression levels is presented relative to GAPDH (for mRNA and circRNA) or U6 (for miRNA) and calculated using the $2^{-\Delta\Delta CT}$ method.

RNA fluorescence in situ hybridization (FISH)

Cells were seeded into a glass-bottom dish and incubated in prehybridization buffer at 37 °C for 30 min, followed by hybridization buffer with Cy3-labeled circPROSC probes (Ribobio, China) in a humid and dark environment at 37 °C overnight. The samples underwent three washes using a heated (42 °C) solution containing 0.1% Tween-20 and 4× saline sodium citrate (SSC), followed by two washes with 2× SSC and 1× SSC at 42 °C. The cell nuclei were stained with 4,6-diamidino-2-phenylindole (DAPI). Images were acquired by confocal microscopy (Zeiss, LSM700, Germany).

CircRNA pull-down

Biotin-labelled circPROSC probe (5′-GAGATCCCATCAATTTGTTG-3) and control probe (5′-ACTCTACGCTCTCACCATCC-3′) (Ribobio, China) were obtained for circRNA pull-down and the assay was performed as previously described[2]. Briefly, after lysis with Thermo Scientific Pierce IP Lysis Buffer (Thermo, USA), sonication, and centrifugation, 50 µl of supernatant was aliquoted for input, and the remaining was incubated with biotin-labelled probes and streptavidin beads (Life Technologies, USA) overnight. The beads were washed with lysis buffer, and proteinase K was used to reverse the crosslinking. Finally, the RNA complexes were extracted with TRIzol for later detection.

Luciferase reporter assay

Luciferase reporters were generated by cloning the circPROSC, mutant circPROSC, wild-type WNT3A-3′UTR, or mutant WNT3A-3′UTR into GP-miRGLO vectors. 293T cells were seeded in 6-well plates to approximately 60% confluence. Subsequently, the cells were cotransfected with luciferase reporter plasmids and miR-6815-5p mimic or NC. After 48 h, the changes in luciferase activity were quantified using the dual Glo luciferase assay system (Promega, USA). Renilla luciferase activities were used as the internal reference.

RNA immunoprecipitation (RIP)

The RIP assay was performed using a Magna RIP kit (Millipore Magna, USA). Briefly, hMSCs were lysed in RIP lysis buffer with RNase and proteinase inhibitors. Magnetic beads conjugated with 5 μg of anti-Argonaute2 (AGO2) antibody (CST, USA) or control IgG (Abcam, USA) were incubated with the RIP lysate overnight at 4 °C. Subsequently, the immunoprecipitated RNA was purified and then detected by qPCR. Relative enrichment was normalized to input samples.

Ectopic bone formation assay

hMSCs were transfected with different lentiviruses (OE-NC, OE-circPROSC, sh-NC, and sh-circPROSC), mixed with 45 mg of β-TCP ceramic particles, incubated in a humidified incubator for 12 h at 37 °C and transplanted into the dorsal surface of 6-week-old nude mice subcutaneously, as described previously[3]. The implants were collected and then fixed in 4% paraformaldehyde at eight weeks postimplantation.

Preparation of siRNA liposomes

To prepare cationic lipid nanoparticles, a mixture of DOTAP, DSPC, cholesterol, and DSPE-PEG2000-COOH was made in a molar ratio of 50:38.5:10:1.5. This mixture was dissolved using a 2:1 chloroform: methanol solution. Using a rotary evaporator under vacuum, the solvents were completely evaporated to form a self-assembled lipid film. The film was then rehydrated with PBS, followed by sonication in a bath sonicator. The siRNA was dissolved in a citrate buffer and rapidly mixed with the lipid mixture by vortexing. Unencapsulated siRNA was subsequently removed by ultrafiltration centrifugation.

Isolation of calvarial mesenchymal stem cell membranes

To analyze the membranes of calvarial mesenchymal stem cells from mice, the cells were first washed three times with sterile PBS and then lysed for 10 minutes in ice-cold Buffer A (P0033, Beyotime, China) supplemented with phenylmethylsulfonyl fluoride (PMSF, ST506, Beyotime, China). The cell suspension was then homogenized 25 times using a glass homogenizer to disrupt the cell membranes. After centrifugation at 700 g for 15 minutes at 4°C, the supernatant is collected and further centrifuged at 14,000 g for 30 minutes at 4°C. The calvarial mesenchymal stem cell membranes are then obtained by collecting the sediment from the bottom.

Biomimetic nanoparticles (MM@Lipo/siRNA)

MM@Lipo/siRNA nanoparticles are obtained through the extrusion method[4]. Briefly, the membrane is mixed with Lipo/siRNA and then sonicated for 3 minutes on an ultrasonicator (FS30D, 42 kHz, 100 W). The mixture solution is then transferred to an extruder (Avestin, LF-1, Canada) and passed through a polycarbonate porous

membrane (200 nm) to harvest the MM@Lipo/siRNA nanoparticles.

General characterization of nanoparticles

Transmission electron microscopy (TEM) was utilized to observe the topographical characteristics of nanoparticles. Dynamic light scattering (DLS, Nano ZS90 Zetasizer, Malvern) was employed to measure the size and zeta potential of the nanoparticles. DiL-labeled liposomes (red fluorescence) were mixed with DiO-labeled mesenchymal stem cell membranes (green fluorescence) and incubated in the dark for 2 h to allow membrane fusion, thereby forming MM@Lipo nanoparticles. The degree of membrane fusion was characterized using a confocal laser scanning microscope (CLSM, Olympus, FV1200, Japan). Membrane proteins from the MM@Lipo nanocomplex were extracted using a membrane protein extraction kit. The protein samples were separated using 10% SDS-PAGE gel and stained with Coomassie Brilliant Blue. Additionally, specific surface markers of MSCs, CD73, CD44, and CD34, were identified by Western blotting. The release behavior of siRNA from Lipo/siRNA and MM@Lipo/siRNA was investigated at pH 7.4. Formulations with an equivalent loaded siRNA dose (5 mg/mL, 1mL) were placed in dialysis bags, immersed in 10 mL PBS (pH 7.4), and stirred in a 37°C water bath. At each time point, 200 μL of the removed solution was replaced with an equal volume of fresh PBS, and the fluorescence spectra of the solution were analyzed.

In vitro interaction with macrophages

For the in vitro immune evasion assay, RAW264.7 cells were incubated with MM@Lipo/siRNA complexes and Lipo/siRNA complexes (each well containing 1 μg of Cy5-labeled siRNA) for 2 hours and 8 hours. After incubation, the nuclei were

stained with DAPI and imaged using a fluorescence microscope.

In vivo targeted therapy

In Twist1$^{+/-}$ mice, coronal suture fusion occurs between postnatal day 9 (PN9) and PN13. Based on previous studies, local intervention at PN1 and PN5 effectively prevents early ossification of the coronal suture[5]. Therefore, PN1 and PN5 were selected to cover both the initial developmental phase and the early pathological onset. In addition, in vivo siRNA delivery studies typically employ a dose range of 40–200 μg/kg to achieve efficient gene silencing with good biocompatibility[6]. Accordingly, we administered MM@Lipo carrying si-circPROSC (100 μg/kg) or control si-NC locally into the coronal sutures at PN1 and PN5. This strategy ensured high local accumulation and effective molecular and histological modulation at a relatively low total dose. Seven days later, the mouse skulls were reconstructed using micro-CT scanning.

Micro-CT analysis

The samples were scanned using μCT 40 (Scanco Medical AG, Switzerland), as described previously[7]. The micro-CT parameters were as follows: electric current, 550 μA; source voltage, 70 kV; and scanning resolution, 18 μm per pixel. The three-dimensional structural parameters, including bone mineral density (BMD) and bone volume fraction (BV/TV), were calculated to compare bone regeneration (n = 6).

Histological staining

The specimens were decalcified in 10% EDTA (pH 7.4) for 1 month, dehydrated, and embedded in paraffin. Subsequently, the embedded sections of 5 μm thickness were

subjected to staining with hematoxylin and eosin (H&E) and Masson's trichrome staining for subsequent histological analysis. Images were obtained with a microscope (TE2000U, Nikon Corp, Japan).

Immunofluorescence staining

The sample slides and cells were fixed in 4% paraformaldehyde for 15 min and washed three times with PBS, followed by permeabilization with 0.1% Triton-X 100 for 10 min. Subsequently, the samples and cells were incubated with anti-RUNX2 or anti-OPN (1:100; Abcam) at 4 °C overnight and were then further incubated with the corresponding secondary antibody as recommended in the dark. After three washes, nuclei were stained with DAPI (Beyotime, China). Finally, fluorescence images were acquired by Zeiss 700B confocal microscope (Zeiss, Germany).

Behavioral assays

Behavioral tests were conducted on WT mice, Twist1$^{+/-}$ mice with bilateral coronal suture fusion, and Twist1$^{+/-}$ mice treated with MM@Lipo/si-circPROSC. All behavioral assessments, including the three-chamber social interaction test, rotarod test, and novel object recognition test, were performed at 6 weeks of age. Prior to each test, mice were acclimated to the behavioral testing room for at least 60 minutes to minimize environmental stress. All behavioral experiments and subsequent data analyses were conducted by experimenters blinded to genotype and treatment conditions.

Novel Object Recognition test

The novel object recognition (NOR) test was conducted to evaluate recognition

memory, as previously described (Leger et al., 2013). The procedure consisted of three sequential phases: habituation, familiarization, and testing. In the habituation phase, each mouse was individually placed in the center of an empty rectangular arena (40 × 40 × 40 cm) and allowed to explore freely for 10 minutes. After 24 hours, two identical objects were placed along the long axis of the arena, 10 cm from the north and south walls. Mice were placed in the center of the arena, facing either the east or west wall, and allowed to explore for 10 minutes (familiarization phase). 3 hour later, one of the identical objects was replaced with a novel object differing in shape and texture but matched in size, and the mouse was placed to the arena for a 10-minute test session (test phase). During the test, the movement trajectory of each mouse was manually recorded. The discrimination index (DI) was calculated as $(T_n - T_f)/(T_n + T_f) \times 100\%$, where T_n and T_f represent the time spent exploring the novel and familiar objects, respectively. The arena and all objects were thoroughly cleaned with 75% ethanol between trials to eliminate olfactory cues.

Three-chamber social interaction test

The three-chamber social interaction test was used to evaluate sociability and social novelty preference in mice. The apparatus consisted of a Plexiglas box divided into three interconnected chambers of equal size, with identical inverted wire cups placed in the side chambers. The test mouse was first placed in the central chamber with the side doors closed for 5 minutes. Then, the side doors were opened to allow free exploration of all three empty chambers for 10 minutes. After this habituation period, the mouse was gently guided back to the center chamber and confined. A stranger mouse (Stranger 1, S1) was placed under a wire cup in one side chamber, and an identical empty wire cup was placed in the opposite chamber. The side doors were

then opened, and the test mouse was allowed to explore freely for 10 minutes. Afterward, the mouse was again returned to the center chamber and confined. A second unfamiliar mouse (Stranger 2, S2) was introduced into the previously empty cup, while S1 remained in place. The test mouse was again allowed to explore for another 10 minutes. The amount of time the test mouse spent sniffing each wire cup was quantified, and the Sociability Index and Social Novelty Index were calculated using the following formulas:

Sociability Index = (Ts1 − Te)/(Ts1 + Te) × 100%;

Social Novelty Index = (Ts2 − Ts1)/(Ts2 + Ts1) × 100%,

where Ts1, Ts2, and Te represent the time spent sniffing Stranger 1, Stranger 2, and the empty cup, respectively. Between each test, the apparatus and wire cups were thoroughly cleaned with 75% ethanol to eliminate olfactory cues from previous subjects.

Rotarod test

The rotarod test was conducted in two phases: training and testing. During the training phase, mice were placed on a rotating rod (Panlab, Harvard Apparatus) set at a constant speed of 4 rpm and were trained to remain on the rod for at least 60 seconds. Twenty-four hours later, the testing phase was carried out. In this phase, the rotation speed was programmed to gradually increase from 4 rpm to 40 rpm over a span of 300 seconds. Mice were positioned on the rod at the starting speed of 4 rpm, and the time taken before falling was recorded as the latency to fall. Each animal underwent three trials per day with 15-minute rest intervals between trials, and testing was repeated across four consecutive days.

Molecular docking

The potential molecular interactions between circPROSC and miR-6815-5p were explored by molecular docking. The circPROSC nucleotide sequence was obtained from circBase, and the mature miR-6815-5p sequence was downloaded from miRBase. Three-dimensional RNA structures were generated using RNAComposer or 3dRNA and saved in PDB format. Non-critical ions and water molecules were removed in PyMOL 2.3.0, and AutoDockTools 4 was used to preprocess circPROSC (receptor) and miR-6815-5p (ligand) by adding polar hydrogens, assigning Gasteiger charges, and saving the processed files in pdbqt format[8]. Molecular docking was performed with AutoDock Vina 1.2.5 using rigid global docking, with the 0.375 Å and the exhaustiveness set to 8[9]. Docking conformations were ranked based on binding free energy (kcal/mol), and the top-ranked pose was selected for further interaction analysis and visualization in PyMOL 2.3.0.

Molecular dynamics simulation

Molecular dynamics (MD) simulations of the circPROSC–miR-6815-5p complex were carried out using GROMACS 2020.3 with the AMBER ff99bsc0χOL3 force field[10]. The top-ranked docking conformation was placed in a cubic simulation box with a minimum distance of 1.0 nm between the complex and the box boundary. The system was solvated with SPC/E water molecules (SPC216 configuration), and Na^+/Cl^- counterions were added to neutralize the system and achieve a physiological salt concentration of 0.15 M. Energy minimization was performed using the steepest descent algorithm, and equilibration was subsequently conducted under the NVT ensemble (500 ps at 300 K) followed by the NPT ensemble (1 ns at 300 K and 1 bar) with position restraints applied to heavy atoms. A 100 ns production MD run was then

performed under periodic boundary conditions using a 2-fs integration timestep. The system temperature was maintained at 300 K with the V-rescale thermostat, and the pressure was controlled at 1 bar using the Parrinello–Rahman barostat. Long-range electrostatic interactions were calculated with the Particle Mesh Ewald (PME) method using a real-space cutoff of 1.0 nm, and all bonds involving hydrogens were constrained with the LINCS algorithm. Trajectory analyses, including root mean square deviation (RMSD), root mean square fluctuation (RMSF), radius of gyration (Rg), hydrogen bond number, and solvent accessible surface area (SASA), were performed using GROMACS utilities. Structural visualization and trajectory inspection were carried out with VMD 1.9.3 and PyMOL 2.4.1. Binding free energy calculations were conducted using the gmx_MMPBSA package.

Surface plasmon resonance (SPR) assay

SPR analysis was performed on a Biacore T200 instrument using an SA sensor chip. Both circPROSC and miR-6815-5p were synthesized by MCE (MedChemExpress, USA). Biotinylated circPROSC was immobilized on Flow Cell 2 (approximately 150–300 RU), with flow cell 1 serving as the reference. The running buffer was HBS-EP+ (10 mM HEPES, 150 mM NaCl, 3 mM EDTA, 0.05% Tween-20, pH 7.4). Serial dilutions of miR-6815-5p (3.125–100 nM) were injected at a flow rate of 30 μL/min, with an association phase of 60–120 s and a dissociation phase of 300 s. After each injection, the chip surface was regenerated with 10 mM glycine-HCl (pH 2.0). Sensorgrams were processed and globally fitted to a 1:1 Langmuir binding model using Biacore Insight evaluation software to obtain kinetic binding parameters.

Immunohistochemistry (IHC)

Formalin-fixed, paraffin-embedded tissues from the heart, liver, spleen, lung, kidney, and brain were sectioned at 4 μm. Slides were deparaffinized in xylene, rehydrated through graded ethanol, and subjected to antigen retrieval by heating in 10 mM sodium citrate buffer (pH 6.0) at 95–100 °C for 15 min, followed by natural cooling to room temperature. Endogenous peroxidase activity was inactivated with 3% H_2O_2 for 10 min, and nonspecific binding was blocked using 5% normal goat serum for 30 min. Sections were incubated overnight at 4 °C with primary antibodies against F4/80 (1:500, Servicebio) or IL-1β (1:500, Abcam). After washing with PBS, an HRP-conjugated polymer secondary antibody was applied for 30 min at room temperature, and signals were visualized using DAB. Slides were counterstained with hematoxylin, dehydrated, cleared, and mounted. Images were captured with an Olympus microscope.

Supplementary Table

Table S1. Upregulated circRNAs with differential expression between healthy control and craniosynostosis

circRNA	FDR	FC	Regulation	chrom	strand	txStart	txEnd	Best transcript	GeneSymbol
hsa_circ_0023397	0.048983898	2.3074306	up	chr11	+	71201890	71202949	NM_018161	NADSYN1
hsa_circ_0001788	0.043300175	2.0365429	up	chr8	+	37623043	37623873	NM_007198	PROSC
hsa_circ_0000803	0.042189483	2.5940376	up	chr17	-	73775150	73775265	ENST00000593254	H3F3B
hsa_circ_0008450	0.038182757	2.9719806	up	chr16	+	66642211	66643906	NM_144601	CMTM3
hsa_circ_0001588	0.037045642	2.2447676	up	chr6	+	26204839	26205043	NM_003545	HIST1H4E
hsa_circ_0076995	0.033878607	2.6384063	up	chr6	-	74228420	74228571	NM_001402	EEF1A1
hsa_circ_0007008	0.033878607	2.3322602	up	chr17	+	7478510	7478576	ENST00000581544	EIF4A1
hsa_circ_0046940	0.032503861	2.1268283	up	chr18	-	10979532	11066221	NM_022068	PIEZO2
hsa_circ_0006623	0.024322094	6.2309476	up	chr8	-	101718878	101719225	NM_002568	PABPC1
hsa_circ_0006667	0.024322094	2.1227868	up	chr3	+	183368083	183390272	NM_017644	KLHL24
hsa_circ_0000945	0.018515837	2.311464	up	chr19	+	48248829	48248993	NM_015710	GLTSCR2
hsa_circ_0004885	0.018515837	2.5570476	up	chr1	-	6659106	6659512	NM_014851	KLHL21
hsa_circ_0003568	0.004017158	7.0620655	up	chr16	+	71954641	71955356	NM_014761	IST1

Table S2. **The binding free energy component of circPROSC with miR-6815-5p was determined on the basis of molecular mechanics Poisson–Boltzmann surface area (MM–PBSA) calculations.**

Energy	Complex
Van der Waals Energy (kJ/mol)	-114.353
Electrostatic energy (kJ/mol)	146.31
Polar solvation energy (kJ/mol)	-140.144
Nonpolar solvation Energy (kJ/mol)	-19.152
Total Binding Energy (kJ/mol)	-127.339

Table S3. Sequences of mimic, inhibitor and shRNA in the study

Gene Name	Sequences (5' to 3')
Lv-shcircPROSC	CAAATTGATGGGATCTCCC
Lv-shNC	TTCTCCGAACGTGTCACGT
mimic-NC F	UUUGUACUACACAAAGUACUG
mimic-NC R	AAACAUGAUGUGUUUUCAUGAC
miR-6815-5p mimic F	UAGGUGGCGCCGGAGGAGUCAUU
miR-6815-5p mimic R	AAUGACUCCUCCGGCGCCACCUA
miR-6815-5p inhibitor	AAUGACUCCUCCGGCGCCACCUA
inhibitor-NC	CAGUACUUUUGUGUAGUACAAA

Table S4. Sequences of primers used for qRT-PCR in the study

**Gene Name**	**Sequences (5' to 3')**
circPABPC1 F	AGTTCGCAATCCTCAGCAAC
circPABPC1 R	TGACTCGTGGAACCTGTGAA
circPROSC F	ACTTCATTGGCCACCTACAGAA
circPROSC R	TCCATAGGCCTCGATCACCAT
mmu-circPROSC F	GGCCATAGGCCTCGATCAC
mmu-circPROSC R	GTCCCGAGATCAAGTGGCA
circCMTM3 F	CTCAAACAAGGGGACTCTGC
circCMTM3 R	AAGTACAAGGCCAGCAGGAA
circKLHL24 F	GATCTATGTTGCCGGTGGAC
circKLHL24 R	TGTTTAGTCTGCGTCCCAAT
PROSC F	ACGATTTTTGGAGAGCGGGA
PROSC R	GTATTCCCTGGCTCAGTGCT
GAPDH F	GGAGCGAGATCCCTCCAAAAT
GAPDH R	GGCTGTTGTCATACTTCTCATGG
Gapdh F	GTCTTCACTACCATGGAGAAGG
Gapdh R	TCATGGATGACCTTGGCCAG
miR-6815-5p F	CGGGCTAGGTGGCGCCGGAGG
miR-6815-5p R	CAGCCACAAAAGAGCACAAT
miR-5002-5p F	CGGGCAATTTGGTTTCTGAGGC
miR-5002-5p R	CAGCCACAAAAGAGCACAAT
miR-1205 F	CGGGCTCTGCAGGGTTTG
miR-1205 R	CAGCCACAAAAGAGCACAAT
miR-3664-3p F	CGGGCTCTCAGGAGTAAAGA

miR-3664-3p R	CAGCCACAAAAGAGCACAAT
miR-4641-3p F	CGGGCTGCCCATGCCATACTTT
miR-4641-3p R	CAGCCACAAAAGAGCACAAT
U6 F	CGCTTCGGCAGCACATATACTAAAATTGGAAC
U6 R	GCTTCACGAATTTGCGTGTCATCCTTGC

Supplementary figures and figure legends

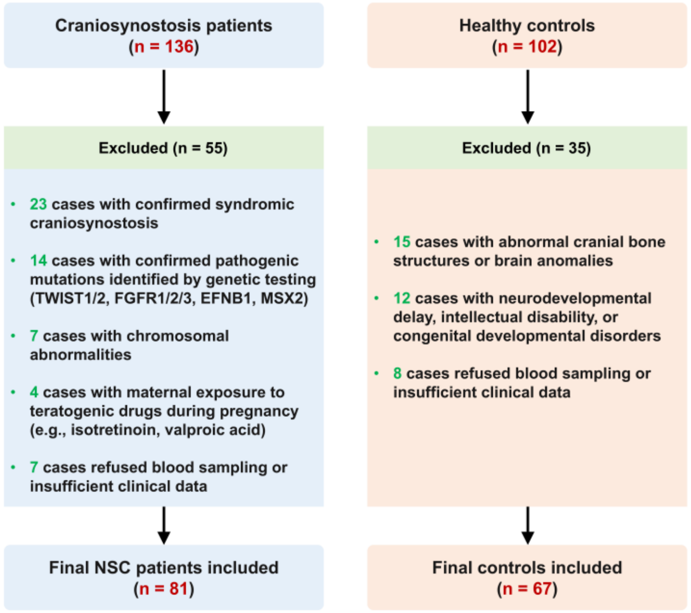

Fig. S1. Flowchart illustrating the inclusion and exclusion process for craniosynostosis patients and healthy controls.

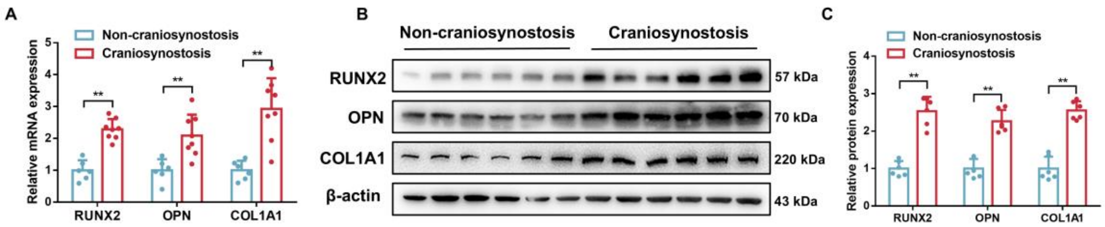

Fig. S2. The expression of osteogenic markers in cranial suture tissues. (A) qRT–PCR analysis of RUNX2, OPN and COL1A1 expression in cranial suture tissues of non-craniosynostosis (n = 6) and craniosynostosis patients (n = 8). (B) Western blots were performed, and (C) relative protein levels of RUNX2, OPN and COL1A1 were determined in the cranial suture tissues of craniosynostosis and non-craniosynostosis. Data are presented as the means ± SD, n = 3 independent experiments. Statistical significance was determined by a two-tailed unpaired t-test. $*P < 0.05$ and $**P < 0.01$.

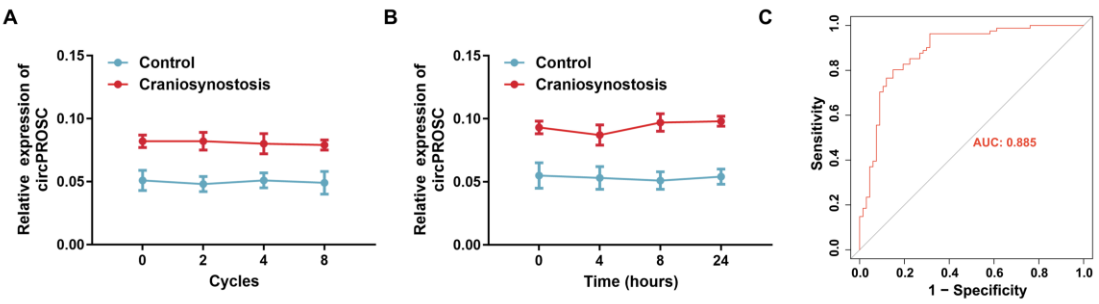

Fig. S3. Plasma circPROSC stability and diagnostic value. (A) The expression of circPROSC was detected after thawing and freezing human plasma repeatedly for 0 cycles, 2 cycles, 4 cycles, and 8 cycles. (B) The expression of circPROSC was detected after placing human plasma at room temperature for 0 h, 4 h, 8 h, and 24 h. (C) ROC curve analysis of plasma levels of circPROSC. Data are presented as the means ± SD, n = 3 independent experiments.

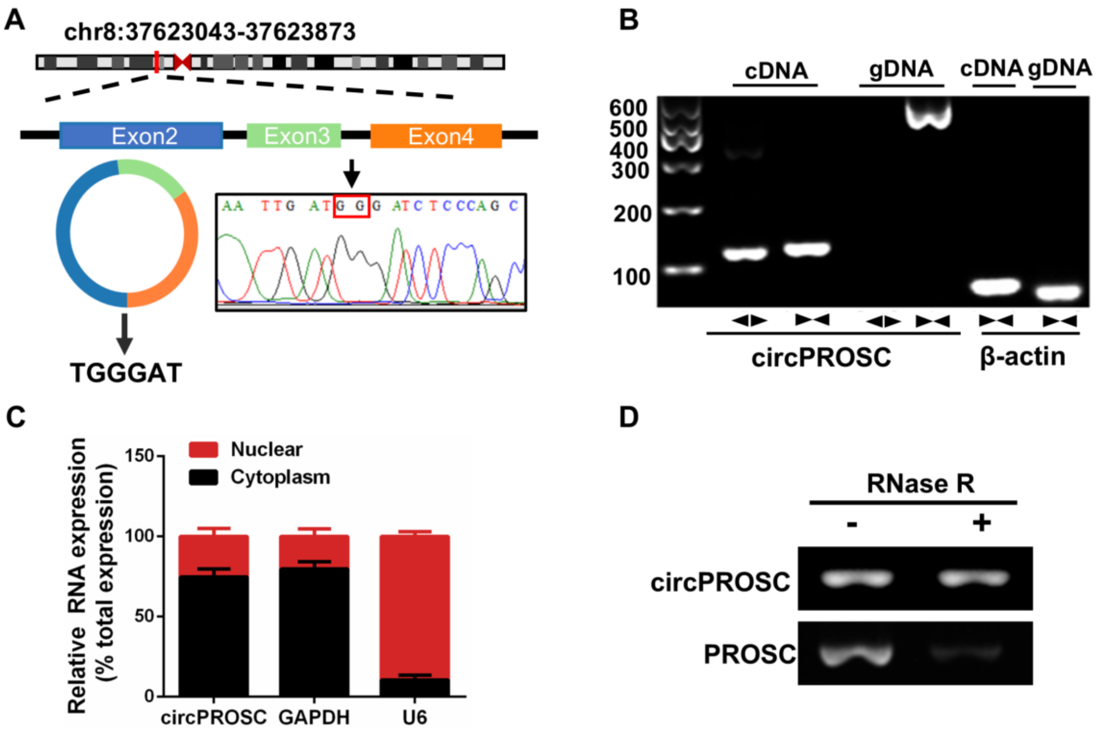

Fig. S4. Characteristics of circPROSC. (A) Schematic of circPROSC formation by the circularization of exons 2, 3 and 4 in PROSC. The back-splice junction site of this circRNA was confirmed by Sanger sequencing. (B) The products amplified using

divergent or convergent primers were verified by PCR. Divergent circPROSC primers amplified circPROSC in cDNA but not gDNA. (C) qRT–PCR of the nuclear and cytoplasmic fractions indicating the expression of nuclear transcripts (U6), cytoplasmic transcripts (GAPDH) and circPROSC. (D) The RNA levels of circPROSC and PROSC were detected by RT–PCR after treatment with or without RNase R. Data are presented as the means ± SD, n = 3 independent experiments.

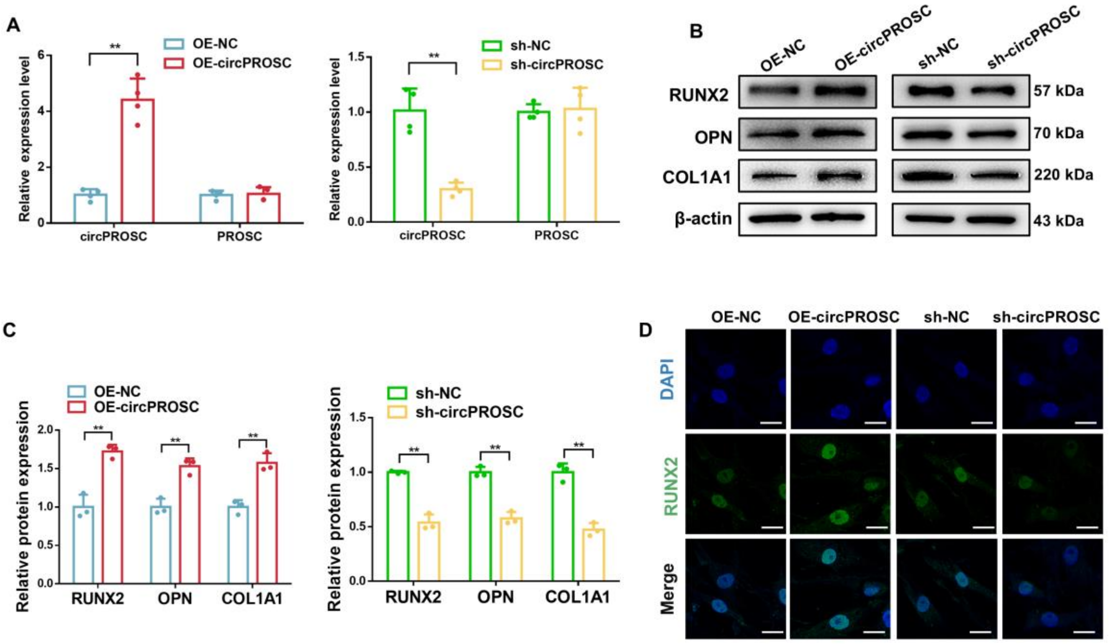

Fig. S5. **CircPROSC increases the expression of osteogenic differentiation proteins.** (A) qRT–PCR analysis of circPROSC and PROSC expression in hMSCs after transfection with circPROSC overexpression and sh-circPROSC lentivirus vectors. (B) Western blots were performed, and (C) relative protein levels of RUNX2, OPN and COL1A1 were detected. (D) Cellular immunofluorescence suggesting the cellular expression levels of RUNX2 on day 7 after osteoblast differentiation. Green represents RUNX2 staining, and blue represents nuclei; scale bar = 20 μm. Data are presented as the means ± SD, n = 3 independent experiments. Statistical significance was determined by a two-tailed unpaired t-test. *$P < 0.05$ and **$P < 0.01$.

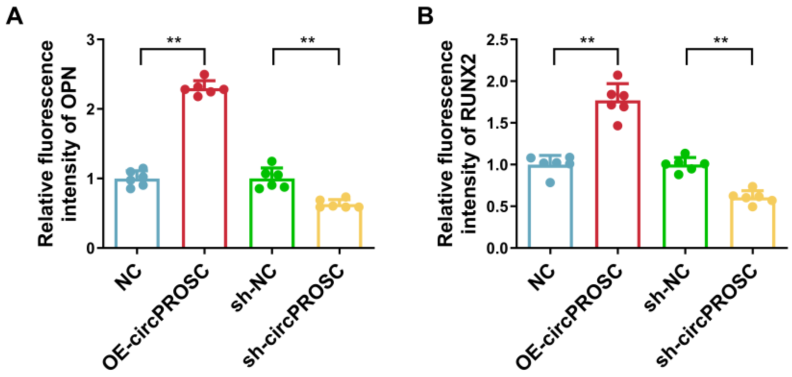

Fig. S6. Immunofluorescence quantification of osteogenic markers in newly formed ectopic bone tissue. (A–B) Relative fluorescence intensity of OPN and RUNX2 was quantified using ImageJ from six biologically independent samples per group (n = 6). Data are presented as mean ± SD. Statistical significance was determined by a two-tailed unpaired t-test. **P* < 0.05 and ***P* < 0.01.

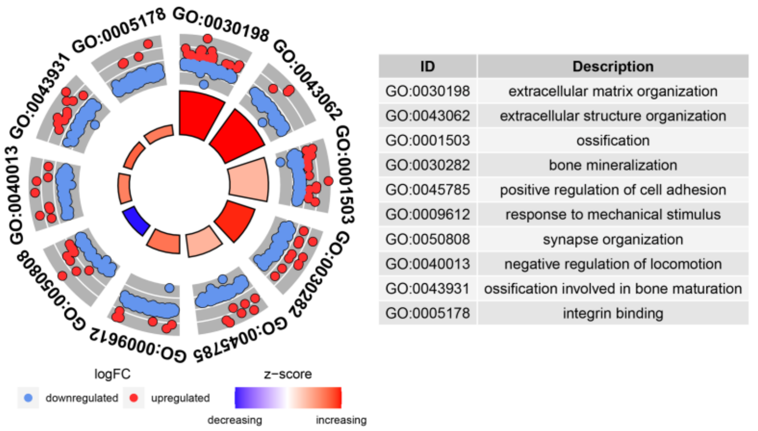

Fig. S7. Enrichment analysis of differential genes after circPROSC overexpression in hMSCs. GOCircle plot of GO enrichment analysis of the differentially expressed genes (DEGs).

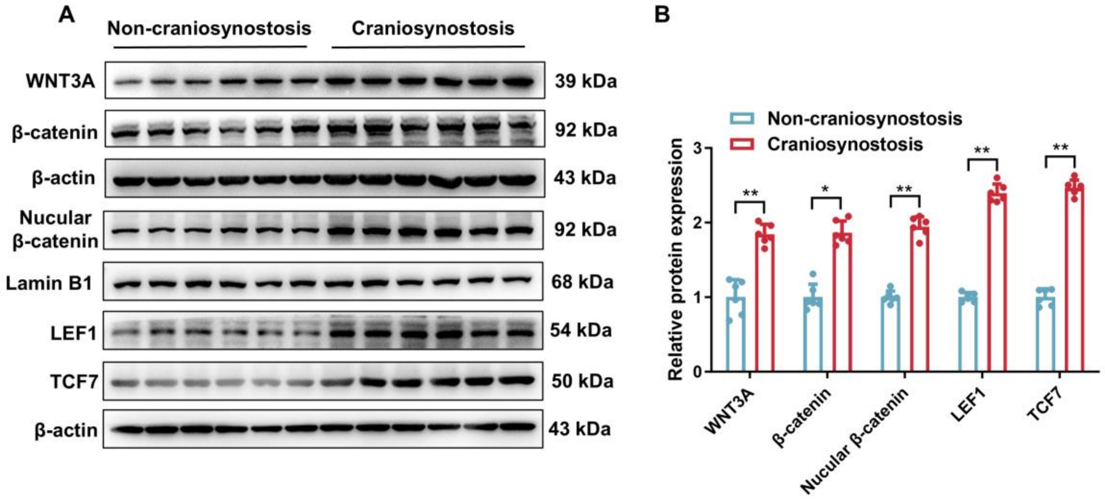

Fig. S8. WNT3A/β-catenin pathway activation in craniosynostosis patient sutures. (A) Western blots were performed, and (B) relative protein levels of nuclear β-catenin, LEF1 and TCF7 were detected. Data are presented as the means ± SD, n = 3 independent experiments. Statistical significance was determined by a two-tailed unpaired t-test. *P < 0.05 and **P < 0.01.

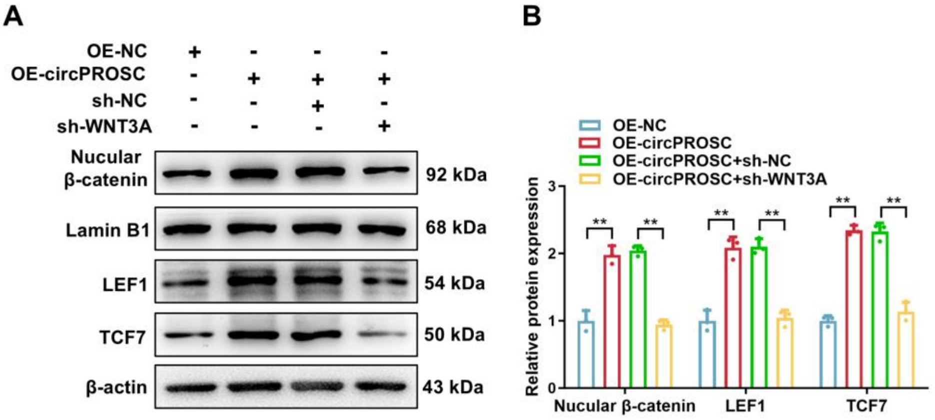

Fig. S9. circPROSC promotes activation of the WNT3A/β-catenin signaling pathway. (A) Western blots were performed, and (B) relative protein levels of nuclear β-catenin, LEF1 and TCF7 were detected. Data are presented as the means ± SD, n = 3 independent experiments. Statistical significance was determined by one-way ANOVA. *P < 0.05 and **P < 0.01.

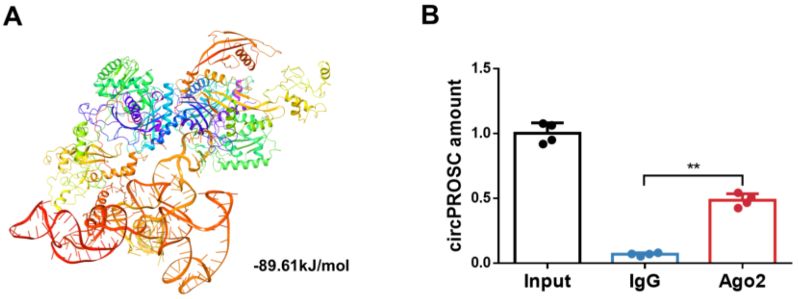

Fig. S10. CircPROSC Interactions with AGO2. (A) Molecular docking of circPROSC with AGO2. (B) AGO2-RIP assay to determine the amount of circPROSC in hMSCs.

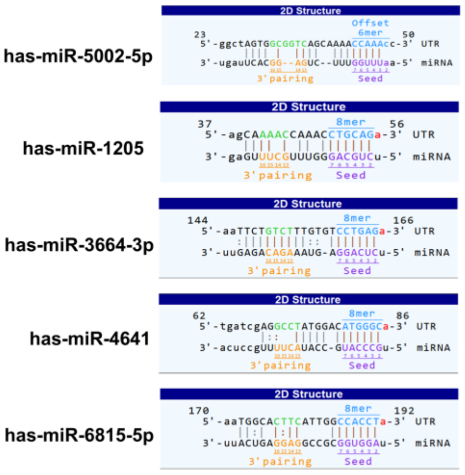

Fig. S11. Prediction of circPROSC binding miRNA by bioinformatics. Predicted miRNA interactions with circPROSC using Arraystar's miRNA target prediction software based on TargetScan and miRanda.

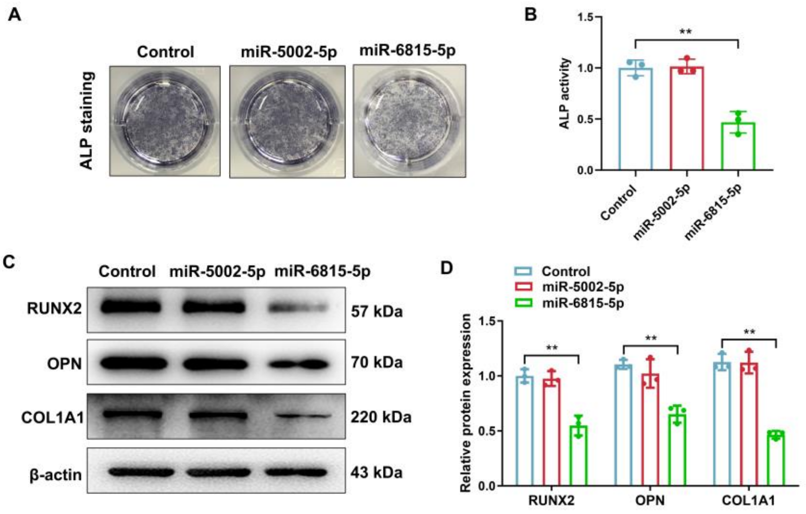

Fig. S12. Effects of miR-5002-5p and miR-6815-5p on osteogenic differentiation of hMSCs. (A) ALP staining of hMSCs was performed on day 7 after osteoblast differentiation. (B) ALP activity was detected on day 7 after osteoblast differentiation. (C) Western blots were performed, and (D) relative protein levels of RUNX2, OPN and COL1A1 were detected. Data are presented as the means ± SD, n = 3 independent experiments. Statistical significance was determined by one-way ANOVA. $*P < 0.05$, $**P < 0.01$.

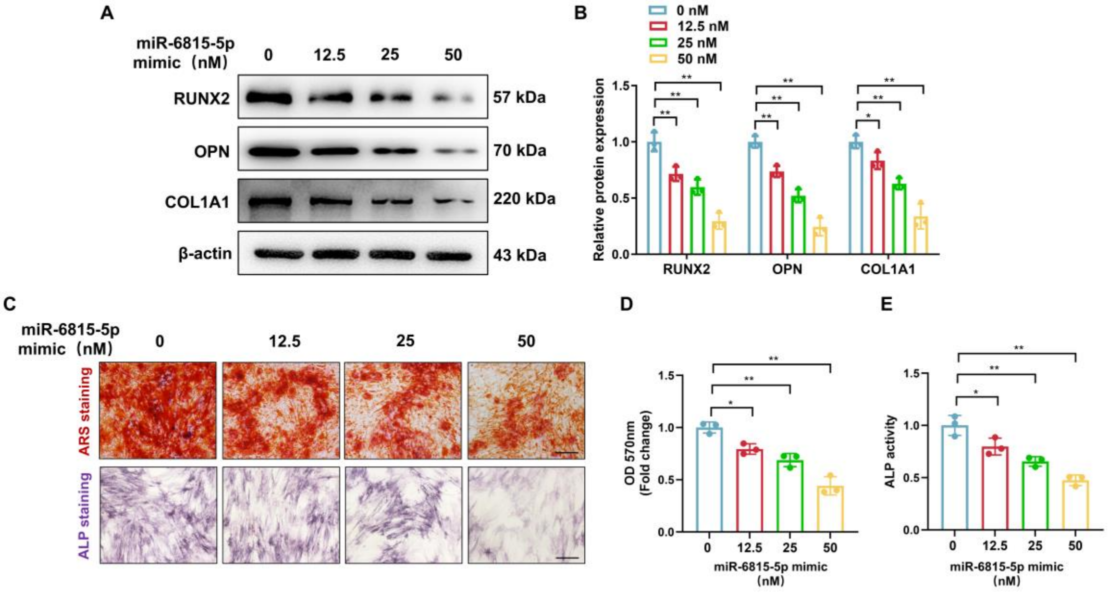

Fig. S13. Effects of miR-6815-5p on osteogenic differentiation of hMSCs. (A)

Western blots were performed, and (B) the relative protein levels of RUNX2, OPN and COL1A1 were determined. (C) ALP content was evaluated by ALP staining and the number of mineralization nodules was evaluated by alizarin red S staining. Scale bar, 200 μm. (D) Semiquantitative analysis of ARS staining (n = 3). (E) ALP activity was detected in hMSCs in the indicated groups (n = 3). Data are presented as the means ± SD; n = 3 independent experiments. Statistical significance was determined by one-way ANOVA. *$P < 0.05$, **$P < 0.01$.

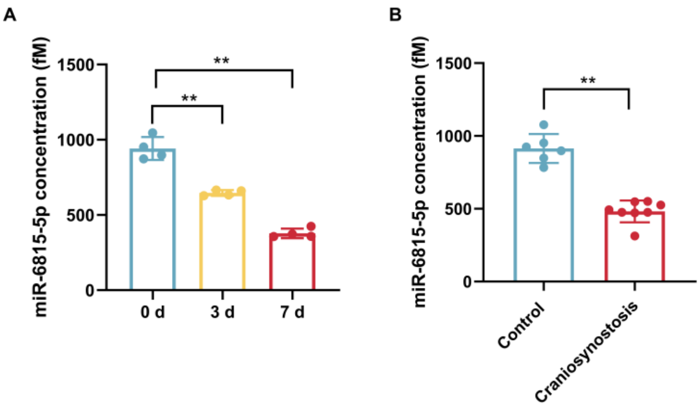

Fig. S14. Physiological concentration of miR-6815-5p. (A) miR-6815-5p levels in hMSCs during osteogenic induction at day 0, day 3, and day 7. (B) miR-6815-5p levels in cranial suture tissues from controls and craniosynostosis patients. Data are shown as mean ± SD, n = 3 independent experiments. Statistical significance was determined by one-way ANOVA (A) and by two-tailed unpaired t-test (B). *$P < 0.05$ and **$P < 0.01$.

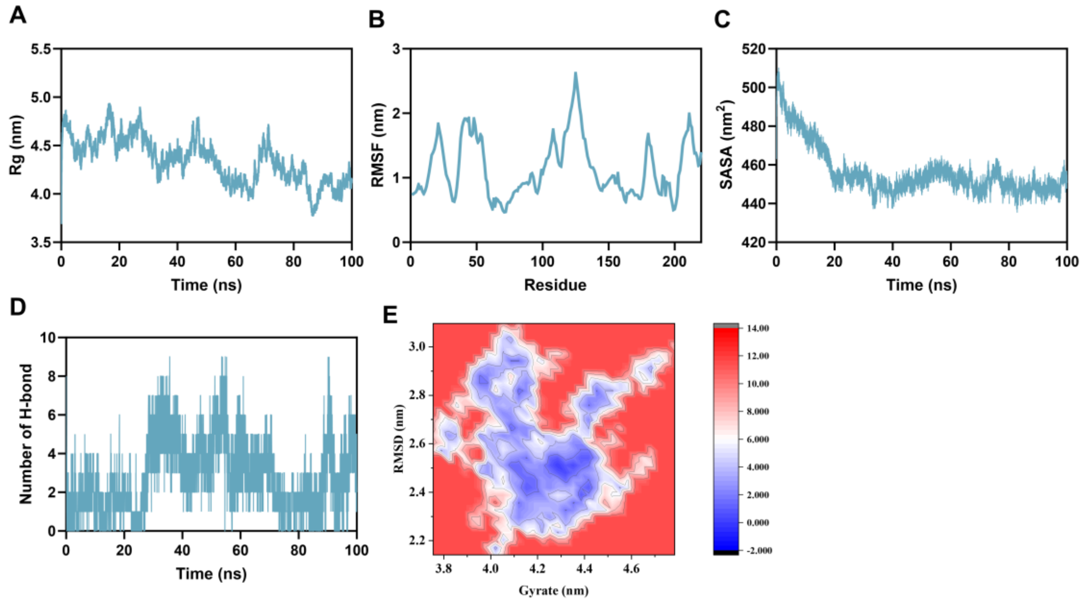

Fig. S15. Molecular dynamics simulations of the circPROSC–miR-6815-5p complex. Dynamic stability and structural properties were evaluated during a 100 ns simulation. (A) Radius of gyration (Rg), reflecting the overall compactness of the complex. (B) Root means square fluctuation (RMSF) of residues, showing local flexibility at the binding interface. (C) Solvent accessible surface area (SASA), indicating conformational compactness. (D) Number of hydrogen bonds between circPROSC and miR-6815-5p as a function of time, reflecting intermolecular interactions. (E) Two-dimensional free Energy Landscape (FEL) of the circPROSC–miR-6815-5p complex, constructed using RMSD and Rg as reaction coordinates.

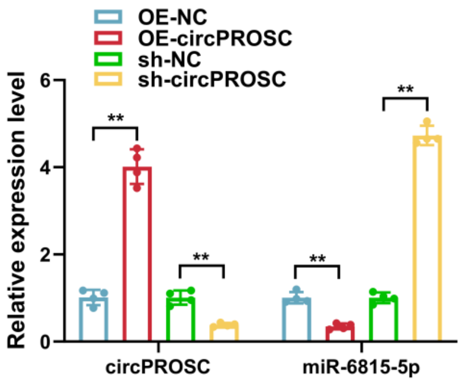

Fig. S16. Effects of circPROSC modulation on circPROSC and miR-6815-5p

expression in hMSCs. Relative expression of circPROSC and miR-6815-5p was detected by qRT-PCR. n = 3 independent experiments. Statistical significance was determined by two-tailed unpaired t-test. **P* < 0.05 and ***P* < 0.01.

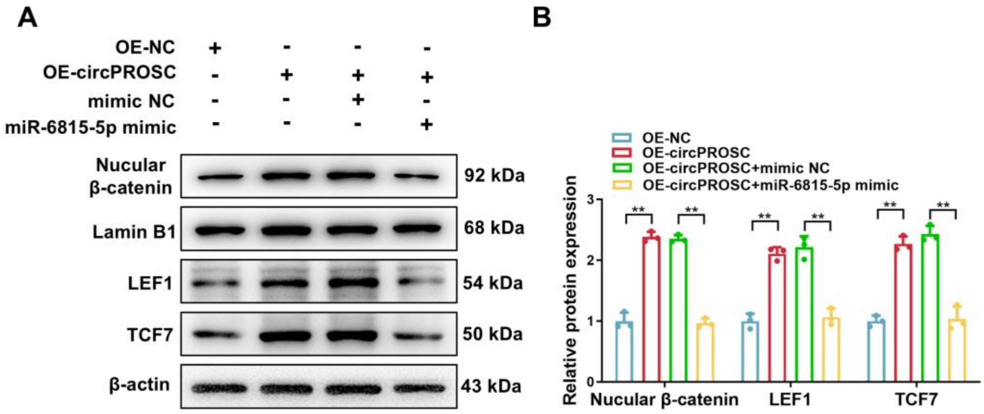

Fig. S17. circPROSC regulates the Wnt/β-catenin signaling pathway via miR-6815-5p. (A) Western blots were performed, and (B) relative protein levels of nuclear β-catenin, LEF1 and TCF7 were detected. Data are presented as the means ± SD, n = 3 independent experiments. Statistical significance was determined by one-way ANOVA. **P* < 0.05 and ***P* < 0.01.

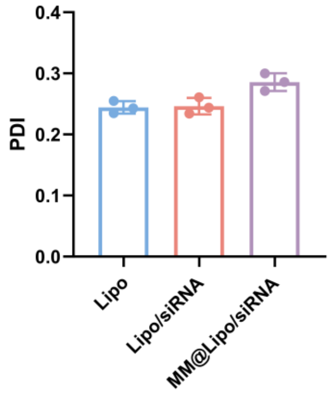

Fig. S18. Polydispersity index (PDI) analysis of different nanoparticle formulations. Data are presented as the means ± SD, n = 3 independent experiments.

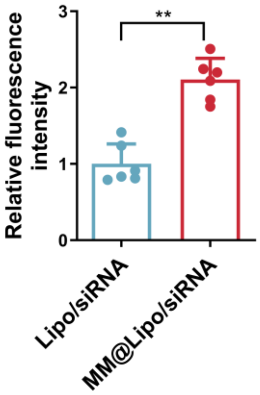

Fig. S19. Quantification of fluorescence intensity in skull. Data are presented as the means ± SD, n = 6 biologically independent mice. Statistical significance was determined by two-tailed unpaired t-test. *$P < 0.05$ and **$P < 0.01$.

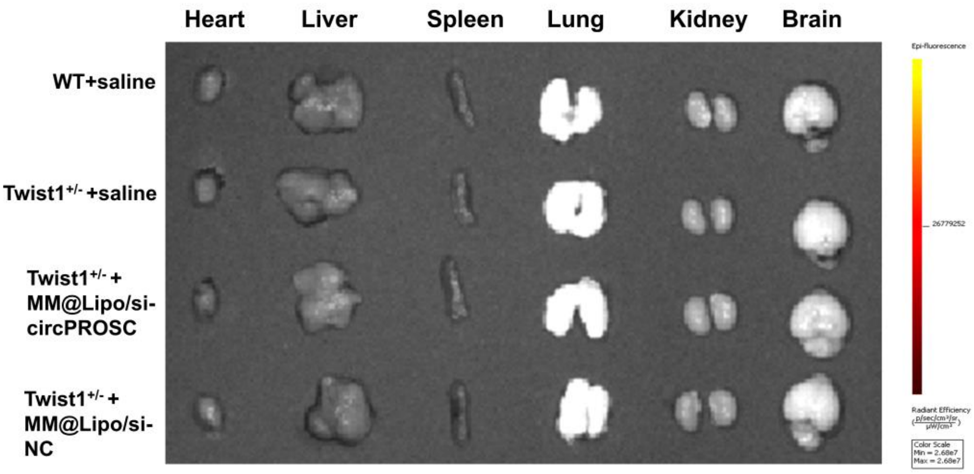

Fig. S20. Fluorescence imaging of the major organs. n = 6 biologically independent mice.

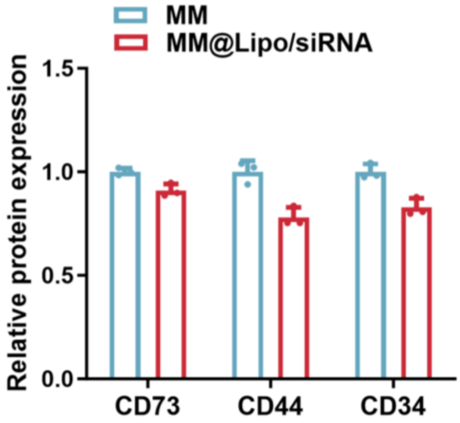

Fig. S21. Relative protein levels of CD73, CD44 and CD34 were detected by Western blotting. Data are presented as the means ± SD. n = 3 independent experiments.

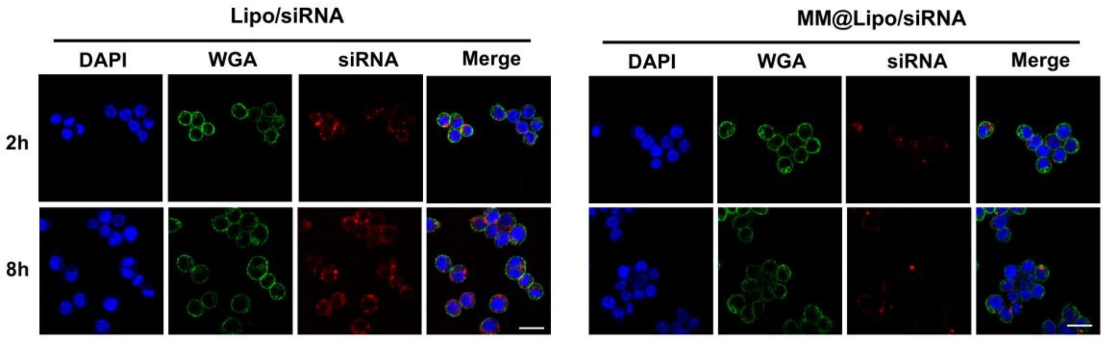

Fig. S22. Immune-escape properties of MM@Lipo/siRNA in vitro. Confocal images of Lipo/siRNA and MM@Lipo/siRNA phagocytosed by RAW264.7 cells. Scale bars = 20 μm.

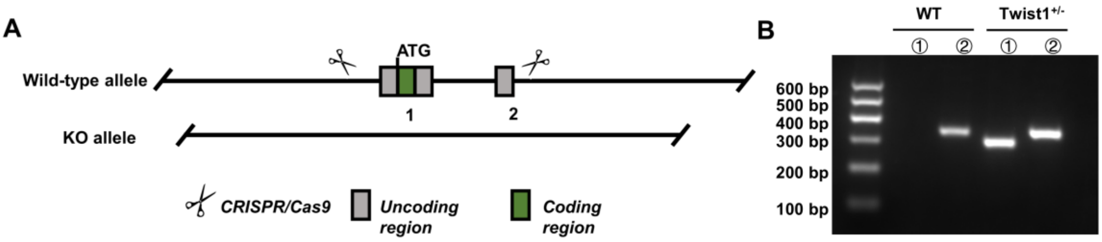

Fig. S23. Genotyping of Twist1 knockout mice. (A) Schematic diagram of Twist1 knockout mice. (B) Genotyping of WT and Twist1$^{+/-}$ mice was performed.

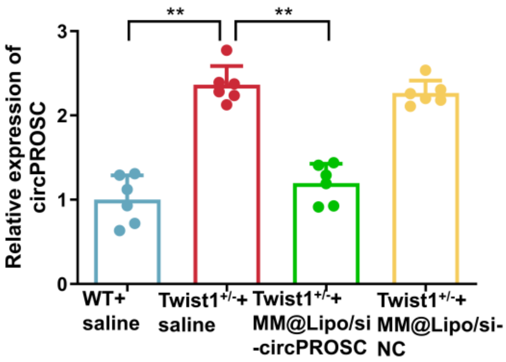

Fig. S24. Relative expression of circPROSC in cranial sutures of mice in different treatment groups. Data are presented as the means ± SD, n = 6 biologically independent mice. Statistical significance was determined by one-way ANOVA. **P* < 0.05 and ***P* < 0.01.

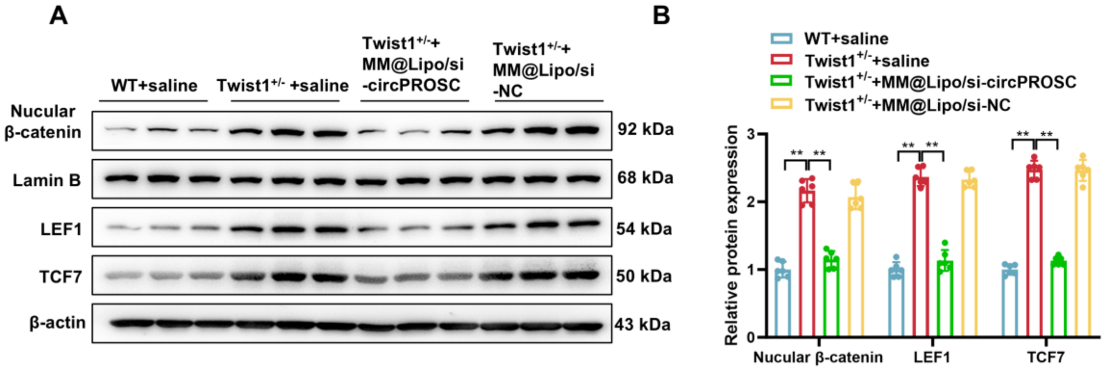

Fig. S25. CircPROSC silencing attenuates WNT/β-catenin pathway activation in Twist1$^{+/-}$ mice. (A) Western blots were performed, and (B) relative protein levels of nuclear β-catenin, LEF1 and TCF7 were detected. Data are presented as the means ± SD, n = 6 biologically independent mice. Statistical significance was determined by one-way ANOVA. **P* < 0.05 and ***P* < 0.01.

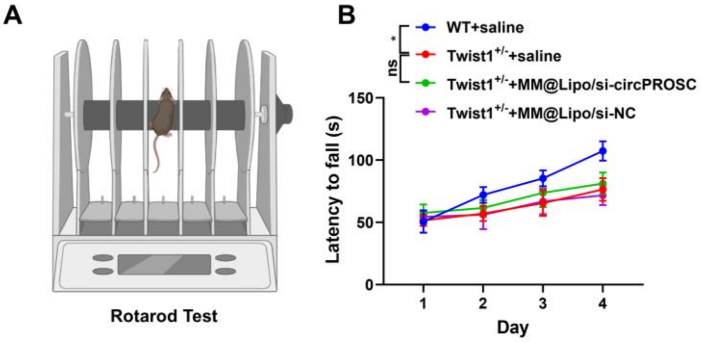

Fig. S26. Evaluation of neurological development and functional outcomes following si-circPROSC treatment in Twist1$^{+/-}$ mice. (A) Schematics of the rotarod test. (B) Rotarod performance scored as time (seconds) on the rotarod (n = 10). Data are presented as the means ± SD, n = 10 biologically independent mice. Statistical

significance was determined by one-way ANOVA. ns $P > 0.05$, *$P < 0.05$ and **$P < 0.01$.

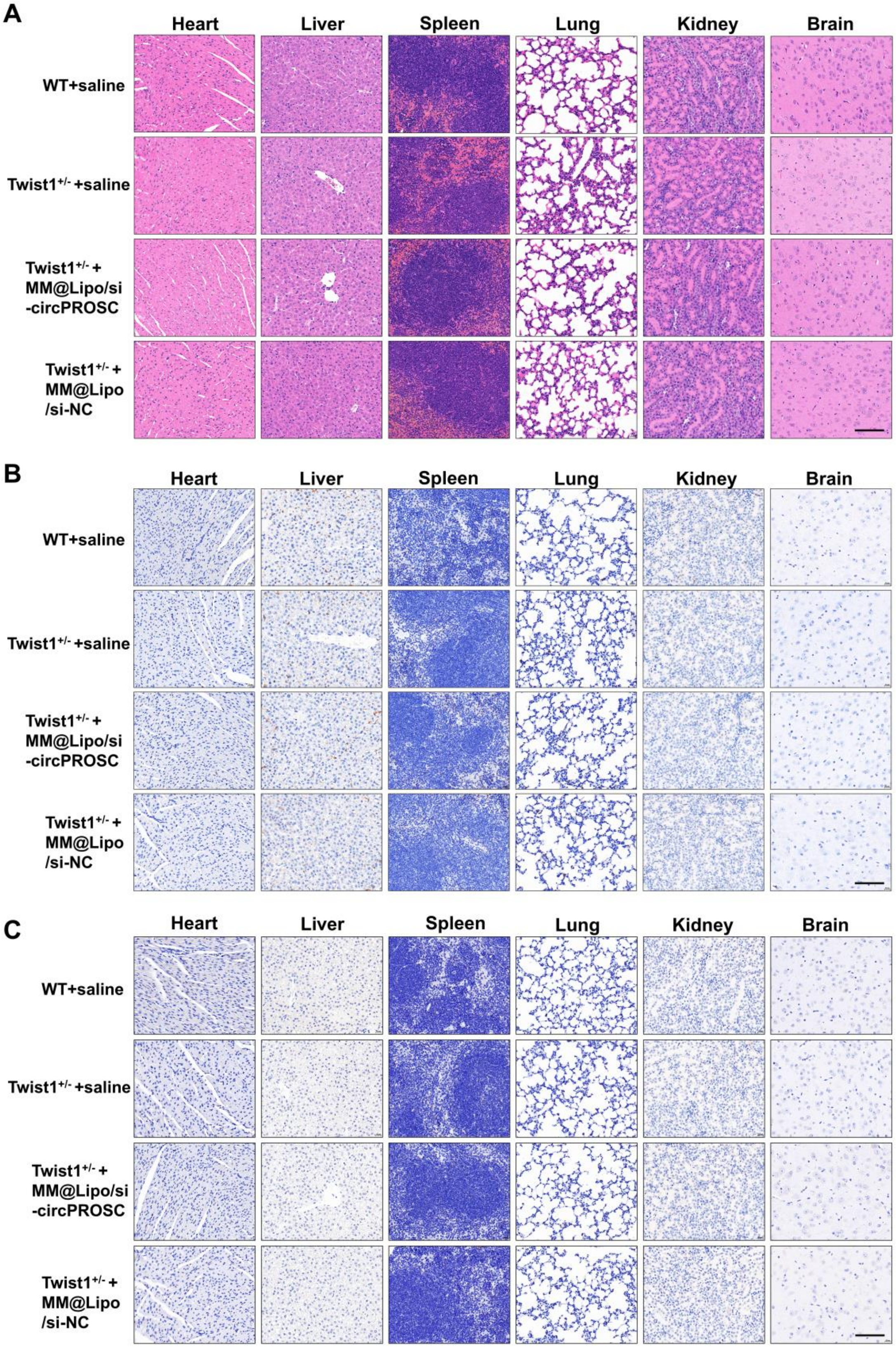

Fig. S27. Biosafety of MM@Lipo/si-circPROSC in vivo. (A) H&E staining images of main organs from mice at PN21, scale bar = 100 μm. Immunohistochemical staining for F4/80 (B) and IL-1β (C) in major organs, scale bar = 100 μm. n = 6 biologically independent mice.

References:

[1] K. L. Burgos, A. Javaherian, R. Bomprezzi, L. Ghaffari, S. Rhodes, A. Courtright, W. Tembe, S. Kim, R. Metpally, K. Van Keuren-Jensen, "Identification of extracellular miRNA in human cerebrospinal fluid by next-generation sequencing," *RNA* **2013**, *19* (5), 712, https://doi.org/10.1261/rna.036863.112.

[2] C. Yang, W. Yuan, X. Yang, P. Li, J. Wang, J. Han, J. Tao, P. Li, H. Yang, Q. Lv, W. Zhang, "Circular RNA circ-ITCH inhibits bladder cancer progression by sponging miR-17/miR-224 and regulating p21, PTEN expression," *Mol Cancer* **2018**, *17* (1), 19, https://doi.org/10.1186/s12943-018-0771-7.

[3] K. Hu, B. R. Olsen, "Osteoblast-derived VEGF regulates osteoblast differentiation and bone formation during bone repair," *J Clin Invest* **2016**, *126* (2), 509, https://doi.org/10.1172/JCI82585.

[4] B. T. Luk, C. M. Hu, R. H. Fang, D. Dehaini, C. Carpenter, W. Gao, L. Zhang, "Interfacial interactions between natural RBC membranes and synthetic polymeric nanoparticles," *Nanoscale* **2014**, *6* (5), 2730, https://doi.org/10.1039/c3nr06371b.

[5] R. J. Tower, Z. Li, Y. H. Cheng, X. W. Wang, L. Rajbhandari, Q. Zhang, S. Negri, C. R. Uytingco, A. Venkatesan, F. Q. Zhou, P. Cahan, A. W. James, T. L. Clemens, "Spatial transcriptomics reveals a role for sensory nerves in preserving cranial suture patency through modulation of BMP/TGF-beta signaling," *Proc Natl Acad Sci U S A* **2021**, *118* (42), https://doi.org/10.1073/pnas.2103087118.

[6] a) C. N. Landen, Jr., A. Chavez-Reyes, C. Bucana, R. Schmandt, M. T. Deavers, G. Lopez-Berestein, A. K. Sood, "Therapeutic EphA2 gene targeting in vivo using neutral liposomal small interfering RNA delivery," *Cancer Res* **2005**, *65* (15), 6910, https://doi.org/10.1158/0008-5472.CAN-05-0530; b) J. Liang, W. Shao, P. Ni, Q. Liu, W. Kong, W. Shen, Q. Wang, A. Huang, G. Zhang, Y. Yang, H. Xin, Z. Jiang, A. Gu, "siRNA/CS-PLGA Nanoparticle System Targeting Knockdown Intestinal SOAT2 Reduced Intestinal Lipid Uptake and Alleviated Obesity," *Adv Sci (Weinh)* **2024**, *11* (40), e2403442, https://doi.org/10.1002/advs.202403442; c) S. Pichu, S. Krishnamoorthy, B. Zhang, Y. Jing, A. Shishkov, B. C. Ponnappa, "Dicer-substrate siRNA inhibits tumor necrosis factor alpha secretion in Kupffer cells in vitro: in vivo targeting of Kupffer cells by siRNA-liposomes," *Pharmacol Res* **2012**, *65* (1), 48, https://doi.org/10.1016/j.phrs.2011.09.001.

[7] J. Du, J. Yang, Z. He, J. Cui, Y. Yang, M. Xu, X. Qu, N. Zhao, M. Yan, H. Li, Z. Yu, "Osteoblast and Osteoclast Activity Affect Bone Remodeling Upon Regulation by Mechanical Loading-Induced Leukemia Inhibitory Factor Expression in Osteocytes," *Front Mol Biosci* **2020**, *7*, 585056, https://doi.org/10.3389/fmolb.2020.585056.

[8] G. M. Morris, R. Huey, W. Lindstrom, M. F. Sanner, R. K. Belew, D. S. Goodsell, A. J. Olson, "AutoDock4 and AutoDockTools4: Automated docking with selective receptor flexibility," *J Comput Chem* **2009**, *30* (16), 2785, https://doi.org/10.1002/jcc.21256.

[9] J. Eberhardt, D. Santos-Martins, A. F. Tillack, S. Forli, "AutoDock Vina 1.2.0: New Docking Methods, Expanded Force Field, and Python Bindings," *J Chem Inf Model* **2021**, *61* (8), 3891, https://doi.org/10.1021/acs.jcim.1c00203.

[10] A. H. Arshia, S. Shadravan, A. Solhjoo, A. Sakhteman, A. Sami, "De novo design of novel protease inhibitor candidates in the treatment of SARS-CoV-2 using deep learning, docking, and molecular dynamic simulations," *Comput Biol Med* **2021**, *139*, 104967, https://doi.org/10.1016/j.compbiomed.2021.104967.
